# Highly Efficient Back-End-of-Line Compatible Flexible Si-Based Optical Memristive Crossbar Array for Edge Neuromorphic Physiological Signal Processing and Bionic Machine Vision

**DOI:** 10.1007/s40820-024-01456-8

**Published:** 2024-07-08

**Authors:** Dayanand Kumar, Hanrui Li, Dhananjay D. Kumbhar, Manoj Kumar Rajbhar, Uttam Kumar Das, Abdul Momin Syed, Georgian Melinte, Nazek El-Atab

**Affiliations:** Smart, Advanced Memory Devices and Applications (SAMA) Laboratory, Electrical and Computer Engineering, Computer Electrical Mathematical Science and Engineering, King Abdullah University of Science and Technology (KAUST), 23955-6900 Thuwal, Saudi Arabia

**Keywords:** Neuromorphic computing, Electrophysiological signal, Artificial vision system, Image recognition, Memristor

## Abstract

The emergence of the Internet-of-Things is anticipated to create a vast market for what are known as smart edge devices, opening numerous opportunities across countless domains, including personalized healthcare and advanced robotics. Leveraging 3D integration, edge devices can achieve unprecedented miniaturization while simultaneously boosting processing power and minimizing energy consumption. Here, we demonstrate a back-end-of-line compatible optoelectronic synapse with a transfer learning method on health care applications, including electroencephalogram (EEG)-based seizure prediction, electromyography (EMG)-based gesture recognition, and electrocardiogram (ECG)-based arrhythmia detection. With experiments on three biomedical datasets, we observe the classification accuracy improvement for the pretrained model with 2.93% on EEG, 4.90% on ECG, and 7.92% on EMG, respectively. The optical programming property of the device enables an ultra-low power (2.8 × 10^−13^ J) fine-tuning process and offers solutions for patient-specific issues in edge computing scenarios. Moreover, the device exhibits impressive light-sensitive characteristics that enable a range of light-triggered synaptic functions, making it promising for neuromorphic vision application. To display the benefits of these intricate synaptic properties, a 5 × 5 optoelectronic synapse array is developed, effectively simulating human visual perception and memory functions. The proposed flexible optoelectronic synapse holds immense potential for advancing the fields of neuromorphic physiological signal processing and artificial visual systems in wearable applications.

**Supplementary Information:**

The online version contains supplementary material available at 10.1007/s40820-024-01456-8.

## Highlights

- A novel optoelectronic synapse compatible with existing chip technology is demonstrated. This device excels in mimicking memory and learning functions using light, making it ideal for future neuromorphic computing in biomedicine.
- Our design surpasses previous models with its ability to switch between short-term and long-term memory states using light pulses.
- Experiments on real-world biomedical data (electroencephalogram, electromyography, electrocardiogram) showed significant improvement in classification accuracy. This highlights the device's potential for advanced physiological signal processing and wearable health monitoring systems.

**Supplementary Information:**

The online version contains supplementary material available at 10.1007/s40820-024-01456-8.

## Graphical abstract

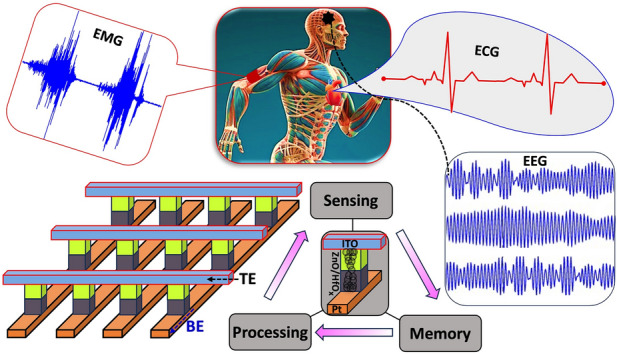

## Introduction

Conventional computing systems based on von Neumann architecture are struggling with significant challenges in terms of high-power consumption and limited information processing speed due to the physical separation of processing and memory units [1]. The human brain, composed of roughly 10^11^ neurons connected by 10^15^ synapses, demonstrates exceptional energy efficiency, fault tolerance, and information transmission capabilities, presenting critical obstacles for the development of next-generation neuromorphic architectures [2–5]. To mitigate the von Neumann bottleneck issue in traditional computing systems, the memristors [6, 7] have excellent capability for use in neuromorphic vision sensors because of that they exhibit great potential by integrating memory, optical sensing, and processing capabilities into a single device [8]. Consequently, image preprocessing and computation in vision sensors can be efficiently executed at the edge, rather than in cloud server systems, resulting in significant reductions in data transfer, latency, and energy consumption [9, 10].

In the current era, artificial intelligence (AI) has greatly contributed to health monitoring and disease diagnosis by utilizing biomedical signals [11, 12]. With the rise of the internet of things (IoT), AI services are moving from the cloud towards edge computing. Edge computing is a decentralized computing model that brings data processing closer to the data source, rather than relying on a centralized server or cloud-based location. Compared with cloud computing, edge computing faces considerable challenges due to large neural network (NN) parameters and hardware resource limitations [13, 14]. Edge computing applications require the effective and efficient integration of both software and hardware components [15–17]. In edge biomedical applications, a universal model usually performs worse than patient specific model due to the variability of individual biomarkers across patients. Inspired by the idea of transfer learning, we present a patient specific method that employs our optical memristive synapse to fine-tune the pre-trained model. In the first stage, we train all the informative features from signals of all available subjects for the general feature extraction. In the second stage, we maintain the feature extraction part with fixed convolutional weights but fine-tune the memristive synapse in fully connected layer through individual patient prediction results or unseen subjects. We evaluate our method with three biomedical tasks: electroencephalogram (EEG)-based seizure prediction, electromyography (EMG)-based gesture recognition, and electrocardiogram (ECG)-based arrhythmia detection. For three physiological tasks with new patients or unseen data, we all observe the improvement in training accuracy with fine-tuning synaptic weights.

While essential for physiological functions, the human visual system's impact extends far beyond, playing a critical role in both survival and learning. In a highly efficient process, the retina swiftly detects light stimuli and pre-processes image information in parallel before the brain undertakes more intricate tasks [18–22]. Recent years have witnessed remarkable advancements in digital vision systems. These systems predominantly rely on conventional technologies such as complementary metal–oxide–semiconductor (CMOS) imagers or charge-coupled device (CCD) cameras [23–26]. They have rapidly evolved to facilitate computer vision through the integration of extended digital processing units, operating in both serial and coarse parallel configurations [27, 28]. Nonetheless, conventional digital artificial vision systems often grapple with challenges such as excessive power consumption, substantial physical dimensions, and prohibitively elevated costs for practical applications. To address these limitations, researchers are now turning their attention to neuromorphic vision sensors, drawing inspiration from biological systems. These sensors seamlessly incorporate image sensing, memory, and processing capabilities, holding the potential to effectively surmount these drawbacks [29–32]. In this regard, photonic memristive devices hold promise for future artificial vision systems, offering not only sensing abilities but also the capacity to perform temporal memory and real-time visual information processing via on-chip computing [33–38].

In order to extend the system performance gains, which is necessary in edge devices, it is important to combine new device technologies with new system architectures which collectively can address the von Neumann architecture wall; the memory wall. One such promising system architecture is based on the ability to integrate different technologies and materials in 3D. While several 3D heterogeneous integration schemes exist which use either 2.5D interposers or through-silicon-vias (TSVs), the monolithic 3D integration approach shows many advantages in terms of ultra-dense integration of different materials, devices and technologies (i.e. logic, memory, sensors, etc.) on random vertical layers within the same chip stack. Nevertheless, the main challenges that the monolithic integration faces are the limitation in the thermal budget (< 450 °C to avoid damaging the previously grown layers and devices) as well as concerns related to the reliability and yield as the number of stacked layers increases. Different solutions are being studied to overcome these challenges such as developing and optimizing growth recipes to achieve layers with high quality materials for the different active layers at lower temperatures, developing back-end-of-line (BEOL) compatible devices and thermally dissipative layers, improving the reliability of the inter-layer vias, etc. [39]

While several previous works have reported BEOL-compatible metal oxides-based single or bilayer flexible memristive devices for data storage and neuromorphic computing applications [40–47], nevertheless, they were fabricated on either PET or PEN organic substrates, or thin mica glass. Moreover, previous studies using oxide based memristors mostly focused on the neuromorphic computing application and not the photo-sensing added functionality or physiological signal processing. Also, most of these reports used the sputter deposition technique to grow the active layers which makes it challenging to achieve ultra-thin uniform layers. Hence, it is still crucial to show a truly back-end-of-line compatible multifunctional integration of photo-sensing, effective information processing, and storage into a single memristive cell on flexible Si substrate for 3D integration within edge devices.

In this study, we present a bilayer BEOL-compatible ultra-thin ZnO (8 nm)/HfO_x_(5 nm)-based multifunctional optoelectronic flexible memristive synaptic device capable of exhibiting electrical and light-induced synaptic features within a single memristive device. Towards making the technology transfer faster and to enable BEOL-compatibility, here, we used a widely employed system in the semiconductor industry—the Atomic Layer Deposition—to grow the sub-10 nm ultra-thin switching layers in the device at low temperatures with excellent uniformity. The device’s electrical synaptic properties are initially evaluated under varying applied voltage pulses. The device successfully demonstrated numerous synaptic features such as long-term potentiation (LTP), long-term depression (LTD), short-term plasticity (STP), and paired-pulse facilitation (PPF) through the application of voltage pulse series. Light-induced long-term plasticity (LTP) can be achieved by adjusting the time interval of optical light pulses. The device effectively performs advanced synaptic functions, including photo synaptic current (PSC), photonic PPF, short-term memory (STM), long-term memory (LTM), and learning-forgetting-relearning processes by tuning the 456 nm wavelength optical light. By modulating light intensities and exposure times, the photonic memristive device can accurately emulate human visual perception and visual memory. These findings demonstrate the photonic memristive synaptic device possesses significant potential for developing human visual perception systems as well as its ability to perform patient-specific physiological signal processing. It should be noted that while various oxides have been reported in the past for application in optoelectronic synapses, these have been limited by either their low endurance or reliability due to the un-optimized growth process, their rigidity and thus incompatibility with flexible IoT wearables, and/or their application in image recognition mainly. It should be emphasized that this is the first demonstration of the potential of ZnO/HfO_x_ based optoelectronic synapses in physiological signal processing applications. Table S1 (Note-S1 in Supplementary Information) shows a comprehensive comparison between previously reported oxides-based memristive synapses and this work [37, 42–65].

## Experimental Section

### Device Fabrication

The proposed device was fabricated on a 4-inch Si wafer. Prior to fabrication, the Si wafer was cleaned using isopropyl alcohol (IPA) and deionized (DI) water, followed by drying with N_2_ gas. Initially, a 250 nm SiO_2_ layer was grown on the Si wafer via plasma-enhanced chemical vapor deposition (PECVD) at 400 °C. Then, the bottom electrode Pt was patterned by optical lithography on the wafer. After Pt, a 5 nm HfO_x_ and 8 nm ZnO as a switching layer were grown by atomic layer deposition (ALD) at 250 °C. Subsequently, a 100 nm ITO top electrode was deposited by sputtering and patterned through a liftoff process to prepare an ITO/ZnO/HfO_x_/Pt memristive crossbar array with the cell size of 10 × 10 µm^2^. The detailed fabrication process flow of the crossbar array can be found in Note-S2.

### Device Flexing

To thin down the devices for physical compliance, a deep reactive ion etching (DRIE) tool was used. A thick photoresist (≈10 μm) was spin-coated on the sample to protect the active devices from the top surface. Next, the sample was turned upside-down on a carrier wafer for etching the backside bulk silicon. The whole back-etching process was divided into multiple steps to make sure that the required thickness was achieved without over-etching. In the first four steps, the sample thickness was reduced from 525 to 100 μm, and the final four steps were used to achieve ≈40 μm with a reduced etch rate. The etch process was carried out at a temperature of − 20 °C, 1500 W ICP power and 60W RF power, 20 mTorr pressure, and 80 sccm SF6 flow. In between each etching step, the actual sample thickness was measured using a physical profilometer to confirm the thickness of the substrate. Once the expected thickness was achieved, the photoresist was stripped using acetone/IPA, and the sample was placed on a semi-circular support for further characterization.

### Characterization and Measurement

The cross-sectional structure and layer-by-layer material composition were analyzed using a high-resolution transmission electron microscope ((FIB lamella fabrication: Helios G4 FIB/SEM (ThermoFischer Scientific), STEM imaging and EDS elemental mapping: ThemisZ S/TEM microscope working at 300 kV (ThermoFischer Scientific)). X-ray photoelectron spectroscopy (XPS) was performed on ZnO and HfO_x_ samples under high vacuum using a Kratos Amicus XPS system, equipped with a monochromatic Al Kα X-ray source operating at 10 kV. The device's electrical characteristics were measured using an Agilent B1500A semiconductor device parameter analyzer. For photoinduced measurements, a visible blue light-emitting diode source (456 nm, Shanghai Dream Lasers Technology) and electronic shutter controller (Newport) were employed.

## Results and Discussion

### Physiological Signal Processing

**Fig. 1:**
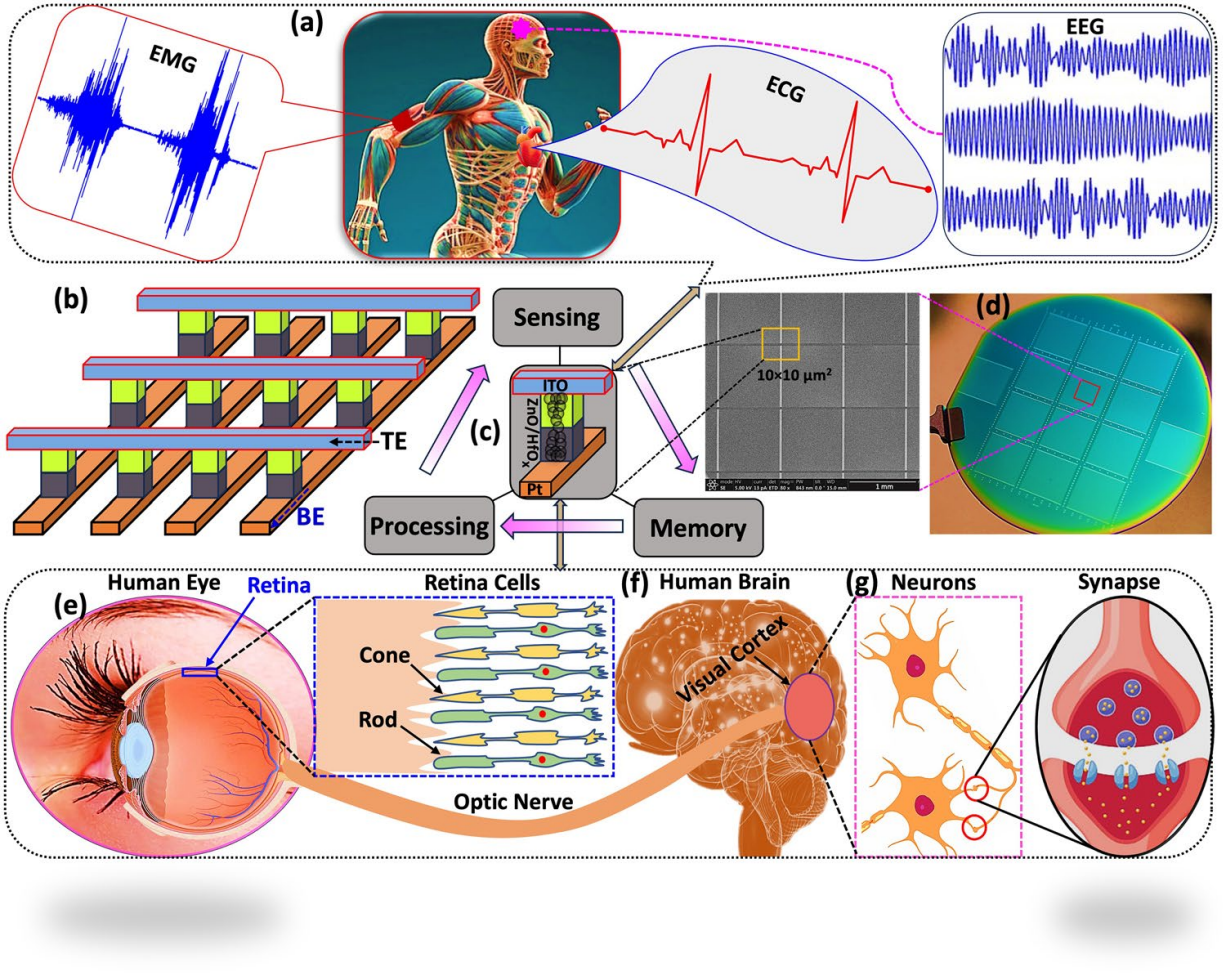
**a** Schematic diagram of physiological signals processing framework which is showing physiological organs such as EEG, EMG, and ECG of human body: Non-invasive EEG signals enable the reconstruction of consciousness patterns in the brain and the detection of eye movements for identity verification, EMG evaluates muscle and nerve function, assisting in diagnosing neuromuscular disorders and muscle-related issues, while ECG records the electrical activity of the heart, aiding in the diagnosis and monitoring of heart conditions like arrhythmias and heart attacks. **b** Schematic architecture of the crossbar array. **c** Integration of visible information sensing-memory-processing of the device. **d** Optical photographic image of the crossbar architecture which was fabricated on 4-inch Si wafer (there are 14 blocks on the wafer, and every block has 100 × 100 crossbar devices) and zoom in SEM image of the crossbar array with the cell size of 10×10 µm^2^. Schematic representation of the biological human visual system which is comprises the retina, optic nerve, and brain. **e** Detailed depiction of the human eye’s retina. **f** Diagram of the human brain. **g** Schematic illustration of neurotransmitter between pre-synaptic and post-synaptic sites in the retina

The human body is a complex organism composed of millions of physiological systems, and as such, physiological activities can serve as indicators of both physical and mental states. The processing of physiological signals plays a critical role in monitoring various aspects of human physiology. Figure 1a shows the schematic diagram of physiological signals processing framework for EEG, EMG, and ECG. The non-invasive EEG signals can be used to reconstruct consciousness patterns in the brain and even detect eye movements for identity verification, EMG assesses muscle and nerve function, aiding in the diagnosis of neuromuscular disorders and muscle-related issues and ECG records the electrical activity of the heart, helping diagnose and monitor heart conditions, such as arrhythmias and heart attacks. These tests play a crucial role in diagnosing and treating a wide range of neurological and cardiac disorders, enabling healthcare professionals to provide targeted care to their patients. However, the effective processing of physiological signals demands attributes like high resolution, sensitivity, speed, and low power consumption, which can pose challenges in practical hardware design. Memristor devices offer a promising solution in this context, demonstrating several advantages over traditional Von Neumann architecture systems in accelerating neural networks. By integrating memristor networks into hardware systems for physiological signal processing, we can achieve higher energy efficiency and lower latency compared to conventional implementations. This innovative approach holds great potential in advancing the field of physiological signal processing. Figure 1b shows the schematic architecture of the ITO/ZnO/HfO_x_/Pt crossbar array. Inspired by biological eyes, our group has developed a ZnO/HfO_x_-based optoelectronic device capable of achieving in-sensor computing. This innovation combines information sensing and neuromorphic computing functionalities within a single device. The opto-electronic device achieves the integration of visible information sensing, memory, and processing, analogous to the functioning of the human visual system, which is depicted in Fig. 1c. Optical photograph of 4-inch Si wafer with integrated of crossbar architecture and zoom in image of high-resolution SEM crossbar array with a single cell size of 10 × 10 µm^2^, as shown in Fig. 1d. Figure 1e shows the human visual system which is composed of the retina, optic nerve, and brain. Retina cells, specifically rods, and cones, capture visual information and convert it into physiological electrical signals. These signals are then transmitted to the brain via the optic nerve. After initial processing in the retina, the brain further processes visual signals to facilitate recognition, learning, and memory functions (Fig. 1f). Information is primarily conveyed from the retina to the brain through the release and reception of transmitters (such as Ca^2+^, Na^+^, or K^+^) between pre-synaptic and post-synaptic neurons across the synapse membrane [66, 67], as depicted in Fig. 1g.

### Materials Characterizations

**Fig. 2:**
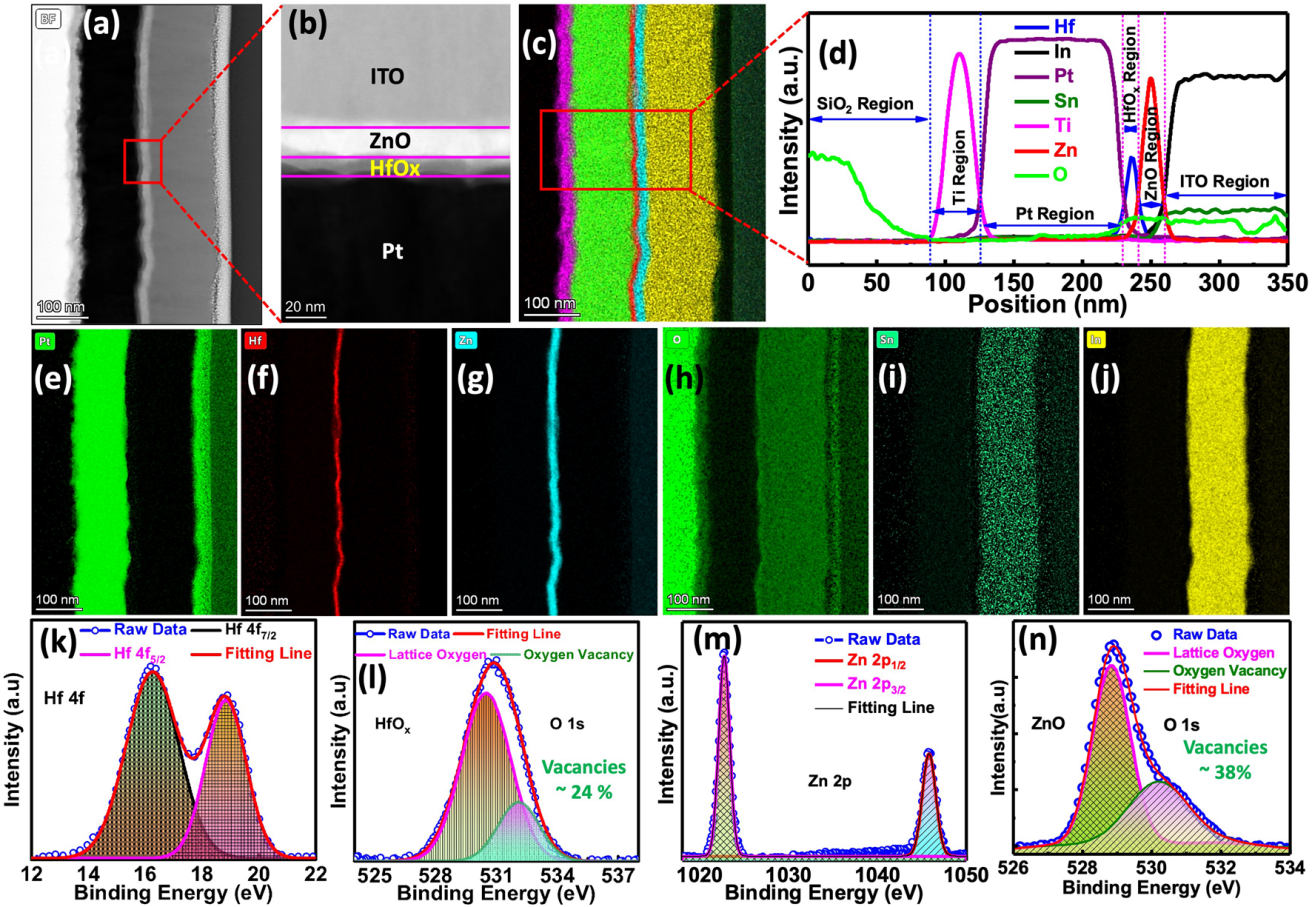
**a**, **b** HR-TEM cross-sectional images of the ITO/ZnO/HfO_x_/Pt device, with scale bars of 200 nm and 20 nm, respectively. **c**, **d** Color TEM and EDS line profiles are used to confirm the presence of various elements within the device. **e-j** EDS elemental mapping for Pt, Hf, Zn, O, Sn, and In within the device. **k**–**n** Depth scan XPS spectra of Hf 4*f*, Zn 2*p*, and O 1*s* peaks in HfO_x_ and ZnO layers

The high-resolution cross-sectional transmission electron microscope (HR-TEM) images of the ITO/ZnO/HfO_x_/Pt device with the scale bar of 100 and 20 nm are depicted in Fig. 2a, b, respectively. Figure 2c, d represents the color TEM image and the energy dispersive spectroscopy (EDS) line profile to verify the thickness of various layers and the elemental profile within the ITO/ZnO/HfO_x_/Pt memristive synaptic device. A uniform ⁓5 nm thick HfO_x_ layer and an ⁓8 nm thick ZnO layers are clearly visible on the Pt bottom electrode. The EDS line profile confirms the presence of different elements, such as indium (In), tin (Sn), oxygen (O), zinc (Zn), hafnium (Hf), and platinum (Pt) within the structure. Elemental mapping for Pt, Hf, Zn, O, Sn, and In supports the existence of a multilayered configuration, as illustrated in Fig. 2e–j.

The chemical composition of the HfO_x_ and ZnO thin films is examined using X-ray photoelectron spectroscopy (XPS) spectra, as shown in Fig. 2k–n. Figure 2k reveals that the core-level XPS spectrum of Hf 4*f* is deconvoluted into two peaks. The Hf 4*f* XPS spectra confirm the doublet peaks at 16 eV (Hf 4*f*_7/2_) and approximately 19 eV (Hf 4*f*_5/2_), corresponding to the Hf–O bonds in the HfO_x_ film [68]. The O 1*s* spectra of the HfO_x_ layer are depicted in Fig. 2l. The major binding energy peak at 530.4 eV, originating from Hf–O bonds in the HfO_x_ film, represents lattice oxygen, while the minor binding energy peak at 532.3 eV is associated with oxygen vacancies in the HfO_x_ layer [69]. Figure 2m presents the XPS spectra of Zn 2*p*, which is deconvoluted into two peaks: the doublet peaks at 1023 eV (Zn 2*p*_3/2_) and 1046 eV (Zn 2*p*_1/2_), corresponding to the Zn–O bonds in the ZnO layer [70, 71]. The doublet O 1*s* spectrum of ZnO confirms that the lower binding energy at 528.5 eV represents lattice oxygen, while the higher binding energy at 530.2 eV signifies oxygen vacancies in the ZnO layer (Fig. 2n) [72]. The core-level XPS analysis of the thin films verifies the distinct presence of HfO_x_ and ZnO layers in the memristive device.

### Electrical RS Characteristics

**Fig. 3:**
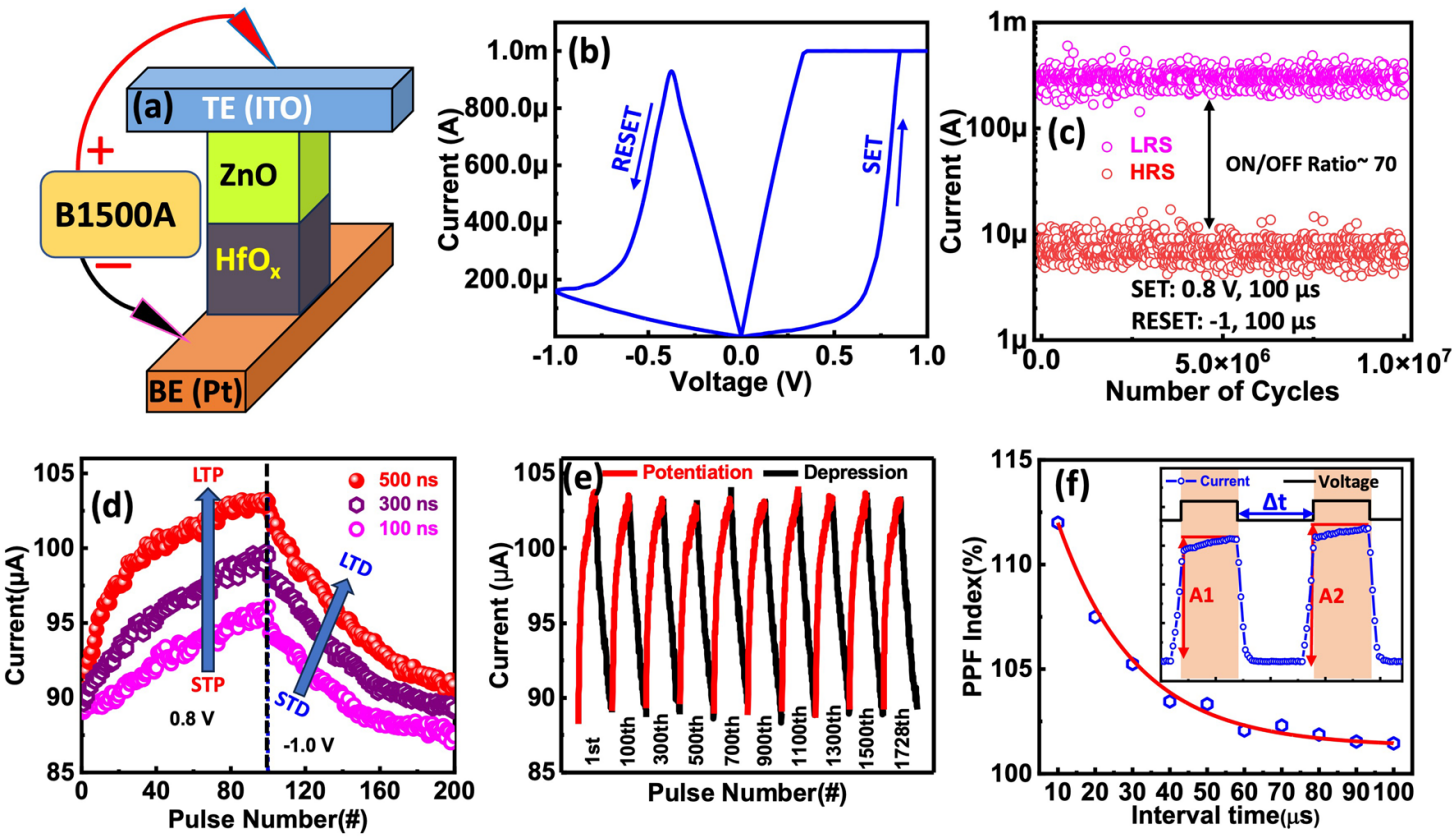
Electrical and synaptic properties of the ITO/ZnO/HfO_x_/Pt device. **a** Schematic representation of the device. **b** Electrical *I–V* characteristics of the memristor, showcasing positive SET (0.7 V) and negative RESET (-0.5 V) transitions. **c** AC endurance of the memristive device at a speed of 100 μs for both SET and RESET operations. **d** Short-term potentiation (STP) to long-term potentiation (LTP) and short-term depression (STD) to long-term depression (LTD) of the memristor, induced by a series of voltage pulses (+ 0.8 V for potentiation, -1 V for depression, pulse width: 100, 300, and 500 ns). **e** Repetitive potentiation and depression 1728 cycles. **f** Paired-pulse facilitation (PPF) index of the device. Inset: schematic depiction of the paired-pulse facilitation measurement

The current–voltage (I–V) and synaptic characteristics of the memristive device were evaluated as shown in Fig. 3. Figure 3a presents a schematic illustration of the ITO/ZnO/HfO_x_/Pt device with a bias applied to the ITO top electrode (TE) and the Pt bottom electrode (BE) grounded. The device exhibits bipolar resistive switching behavior with positive SET (~ 0.7 V) and negative RESET (~ − 0.5 V) transitions, as displayed in Fig. 3b. The electrical forming process of the device is provided in Note-S3. The device exhibits exceptional AC endurance, maintaining stability over more than 10^7^ cycles without any breakdown. It operates with a write voltage of 0.8 V, an erase voltage of − 1 V, and a pulse width of 100 µs. The cumulative distribution functions (CDF) of the *I*_on_ and *I*_off_ states were analyzed, revealing that the variabilities in the on and off states remained within acceptable limits throughout the endurance testing [73–76]. The high stability and prolonged AC endurance are attributed to the conduction mechanism of the device, which is detailed in Note-S4. This mechanism likely involves stable filamentary paths or other reliable charge transport processes that prevent degradation over extended cycling. The device's ability to maintain consistent performance under these conditions underscores its potential for long-term use in various electronic applications, where reliability and endurance are critical [73–77]. The high stability and long-cycle AC endurance of the device are explained based on the conduction mechanism in Note-S4. The DC endurance, device-to-device stability, cycle-to-cycle uniformity, and high-temperature retention tests for the device are detailed in Note-S5.

To emulate bionic synaptic plasticity, voltage pulses were applied to the memristive device, as shown in Fig. 3d. Consistent STP, LTP, STD, and LTD were achieved using 100 identical positive voltage pulses (0.8 V/100 ns, 300 ns, 500 ns) followed by 100 identical negative voltage pulses (− 1 V/100 ns, 300 ns, 500 ns). These results verify that the synaptic weight (conductance) of the device can be increased or decreased by applying positive and negative voltage pulses, respectively. The short-term and long-term potentiation and depression behaviors are demonstrated when sequential voltage pulses are introduced to the pre-synapse, as shown in Fig. 3d. Notably, a longer pulse width generates a relatively fast potentiation process, whereas short-term depression is induced by negative pulse sequences. Similarly, a shorter pulse can lead to a relatively slow depression process. The memristors exhibit long-term potentiation and depression under positive and negative pulse sequences, respectively, suggesting their potential applications in emulating the excitation and inhibition of biological synapses. Figure 3e shows the repeatability of the LTP and LTD performance of the device over a total of 1728 cycles, where each cycle comprises 200 conductance states (100 for potentiation and 100 for depression) corresponding to the applied AC pulses.

Paired-pulse facilitation (PPF) is another crucial aspect of synaptic plasticity that plays a vital role in achieving advanced learning and memory, as illustrated in Fig. 3f. In the inset of Fig. 3f, the PPF index is defined by the expression (A_2_/A_1_) × 100%, where A_1_ and A_2_ represent the current responses of the synapse to the first and second pulse voltage, respectively. Figure 3f illustrates the PPF index as a function of Δt. The PPF index exhibits an exponential decay as Δt increases, characterized by a decaying time constant. This exponential decay mirrors the behavior observed in biological synapses. After applying a voltage on presynaptic terminal, the change in excitatory current is denoted as A_1_. The increase in current for the second pulse, as a postsynaptic pulse in a neuron, is termed as the excitatory post-synaptic current (EPSC) [51]. The increase in current after applying a postsynaptic spike is represented as A_2_.

### Optoelectronic Characteristics

**Fig. 4:**
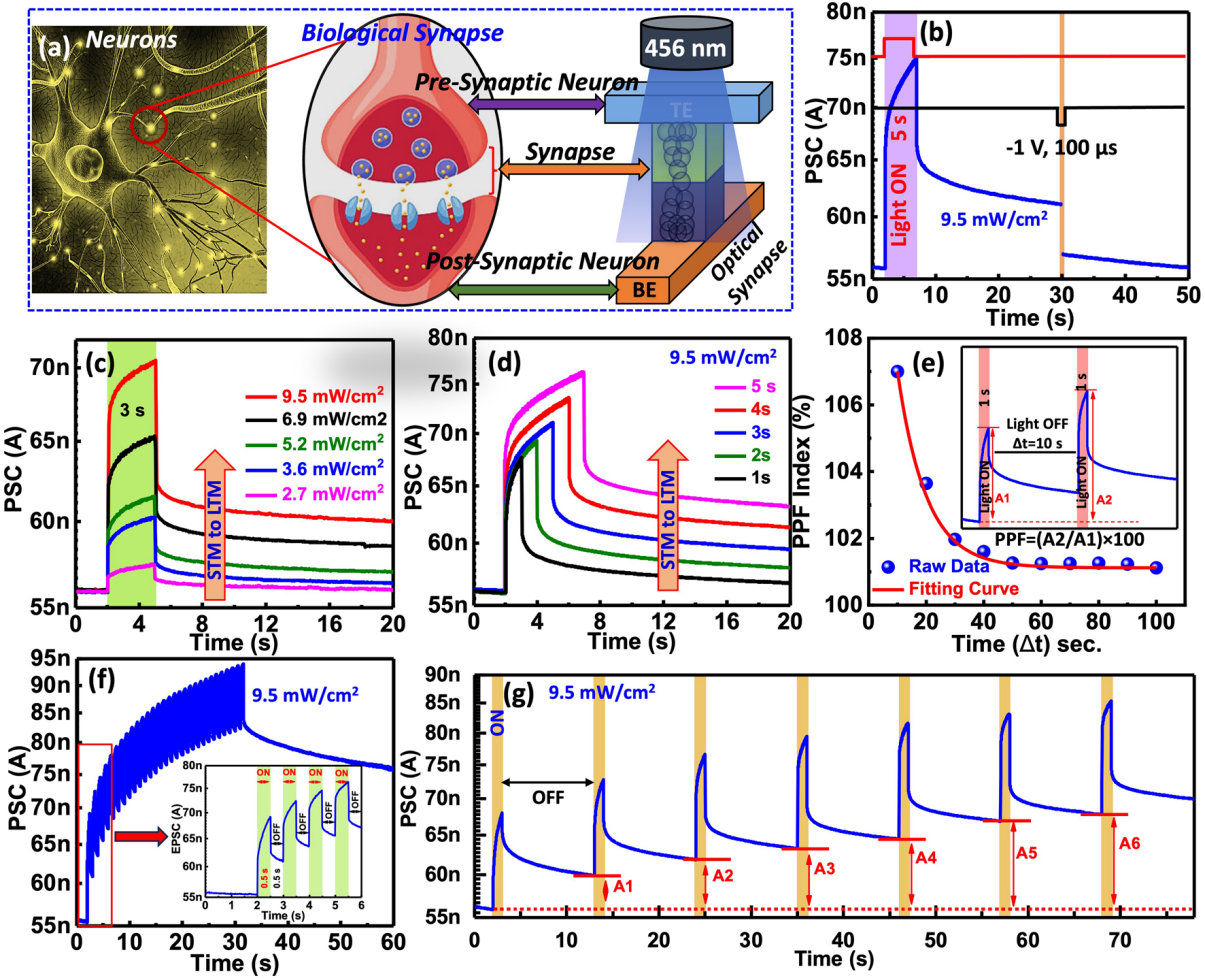
**a** Schematic representation of biological synapse and artificial optoelectronic memristive synapse. **b** Light-induced photonic potentiation using a single blue light (wavelength: 456 nm, light intensity: 9.5 mW cm^−2^, duration: 5 s, indicated by the purple-colored area), and electrical erasure using a voltage pulse (amplitude: − 1 V, duration: 100 µs) in the optical memristive device. **c** Photocurrent response under light for 3 s (indicated by the coral-colored area) at various light intensities (dark, 2.7, 3.6, 5.2, 6.9, and 9.5 mW cm^−2^), followed by photocurrent decay when the light is turned off. **d** Photocurrent response under a light intensity of 9.5 mW cm^−2^ with different time durations (1, 2, 3, 4, and 5 s), followed by photocurrent decay when the light is turned off. **e** PSC of the device for PPF index variation with the time interval (Δt) of photonic pulse pairs. Inset: PSC under blue light (intensity: 9.5 mW cm^−2^, duration: 1 s) pulse pairs with a 10 s time interval. **f** Photocurrent response under consecutive light pulses. Inset: zoom view of. **g** Learning-forgetting-relearning process over seven cycles

In addition to the electrical synaptic properties, we show that our device also demonstrates changes in PSC when exposed to light. Here, we investigate the optoelectronic synaptic features of the device using a series of optical light pulses with varying intensities and durations, as illustrated schematically in Fig. 4a. In the human brain, pre-synaptic neurons connect to post-synaptic neurons. Our two-terminal optoelectronic memristive synapse resembles the biological synapse in the human brain. Figure 4b shows the photo-synaptic current (PSC) response of the device when exposed to optical light (456 nm) at 9.5 mW cm^−2^ for 5 s. All the photo synapses depicted in Fig. 4 were measured under a read voltage of 10 mV and read voltage-dependent responses were depicted and discussed in Note-S6. The PSC of the device increased to 75 nA, with a noticeable current change of 18 nA. This significant increase in PSC is attributed to the photoconductive effect of ZnO and HfO_x_ within the device. After removing the optical light, the device exhibits a gradual decay in PSC instead of quickly returning to the initial current, which could be attributed to persistent photoconductivity (the device's mechanism is depicted in Note-S7) [25]. This PSC decay can be disrupted by applying negative voltage pulses. As shown in Fig. 4b, the decay current of the device drops abruptly from 75 to 57 nA (initial state) when a − 1 V voltage pulse with a 100 µs duration is applied at 30 s, confirming photonic potentiation and electrical depression (elimination).

The PSC of the memristive device is altered by applying various intensity levels (2.7, 3.6, 5.2, 6.9, and 9.5 mW cm^−2^), as shown in Fig. 4c. Greater intensity corresponds to increased current conductance, and its decay is also influenced by light intensity. Lower intensity light results in a minor increase in current conduction, which quickly returns to its initial state upon power removal, resembling STM in the human brain. When higher intensity light is removed, the photocurrent decays slowly and remains at an elevated level above its initial state, acting like LTM in the human brain. Moreover, we examined the transition from STM to LTM by adjusting the illumination duration (1, 2, 3, 4, and 5 s) of light, as presented in Fig. 4d. Longer light exposure times correspond to increased PSC and a slower decay rate. In summary, the optoelectronic memristive device's PSC response can be converted from STM to LTM by modulating light intensity or exposure time, highlighting its efficient ability to mimic advanced memory functions for visible light. We further investigate the light-irradiated synaptic plasticity (PPF) of the optoelectronic device, as shown in Fig. 4e. The photonic PPF was examined when the time interval (Δt) between two identical optical pulses was varied. The photocurrent (A_2_) of the device after the second illumination with an intensity of 9.5 mW cm^−2^ (duration: 1 s, interval: 10 s) is higher than the photocurrent (A_1_) after the first illumination with the same light intensity, which is closely related to Δt. The photonic PPF can also be calculated using the same equation as electrical PPF, as shown in Fig. 3f. The curve demonstrates excellent mimicry of synaptic photonic PPF features, which is an essential synaptic function in neuromorphic vision systems [78].

By continuously switching the light on and off, the device’s PSC significantly improves with increased light pulses, as illustrated in Fig. 4f. The device’s PSC increased from 57 to 93 nA after 30 continuous on/off cycles. This exceptional repeatability in PSC response facilitates the simulation of advanced synaptic functions in synaptic plasticity, represented by the "learning-forgetting-relearning" process [79]. We also demonstrated the learning-forgetting process for our optoelectronic memristor by continuously toggling the light on and off, as shown in Fig. 4g. Turning on the light represents learning and relearning behavior in the device, while turning it off showcases the forgetting behavior. The PSC of the device increases with light exposure and then decreases to an intermediate level after a certain period, suggesting that the learned information is gradually forgotten over time. After continuously repeating the learning or relearning process, the device's PSC improves slightly (A_6_ > A_5_ > A_4_ > A_3_ > A_2_ > A_1_), signifying that previously learned information can substantially enhance memory capacity. The device’s PSC reaches its maximum level (A_6_) after six consecutive learning and relearning processes, indicating the transition from STM to LTM is accomplished in the optoelectronic memristive device.

### Reliability Under Device Flexing

**Fig. 5:**
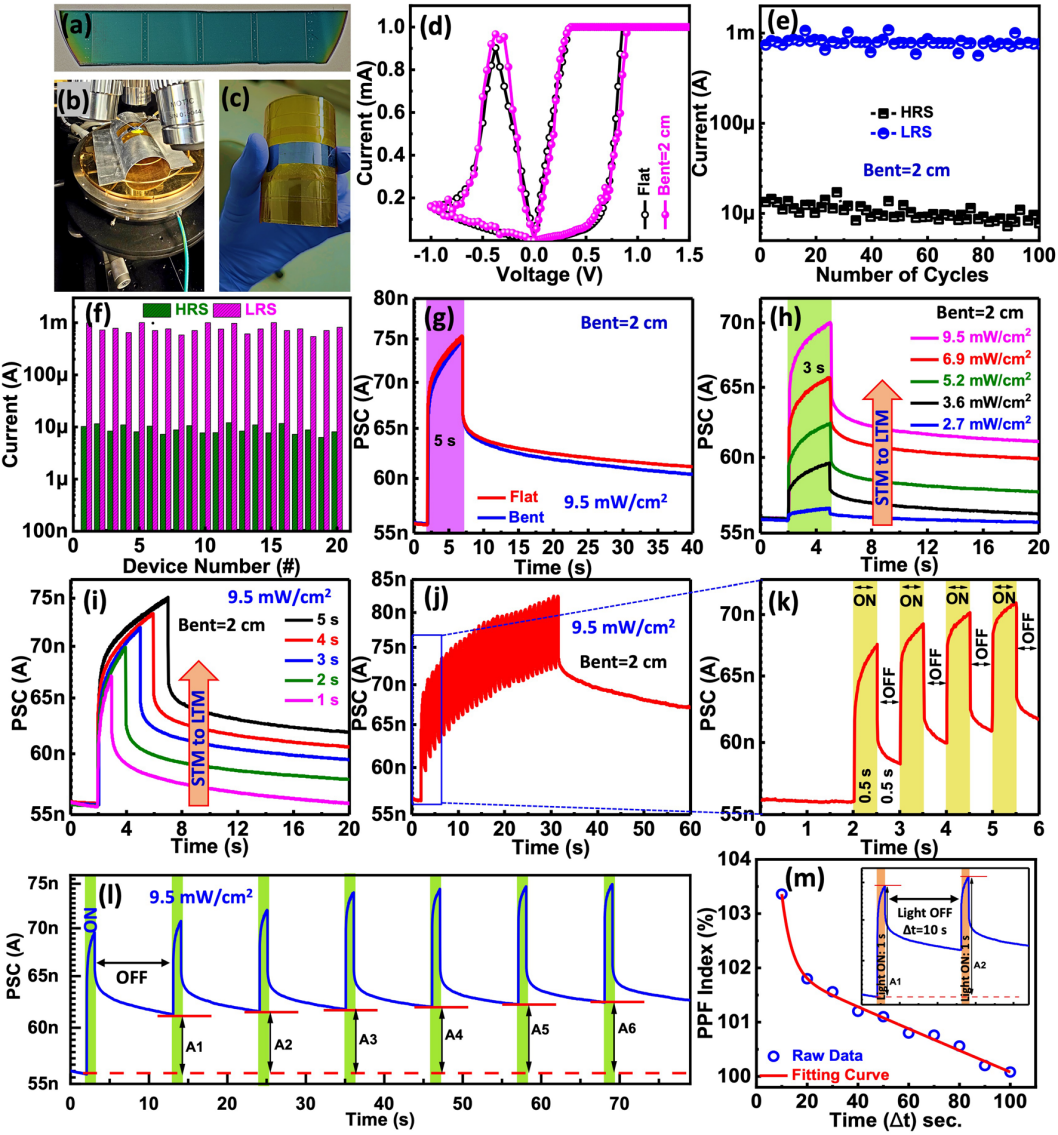
Fig. 5 **a** Photograph of fabricated arrays, **b** photograph of the flexible photonic memristive synapse during optical measurements, and **c** photograph of the flexing of the device. **d** RS characteristics though I-V sweep under flat and bent conditions (2 cm). **e** Endurance characteristics under mended conditions measured up to 100 cycles. **f** Device variability of the fabricated devices under bending conditions in on and off states of the devices. **g** PSC of the device using a single blue light during flat and bending conditions (wavelength: 456 nm, light intensity: 9.5 mW cm^−2^, duration: 5 s, indicated by the purple-colored area). **h** Photocurrent response under light for 3 s (indicated by the green-colored area) at various light intensities (2.7, 3.6, 5.2, 6.9, and 9.5 mW cm^−2^), followed by photocurrent decay when the light is turned off. **i** PSC under a light intensity of 9.5 mW cm^−2^ with different time durations (1, 2, 3, 4, and 5 s), followed by photocurrent decay when the light is turned off. **j** Photocurrent response under consecutive light pulses. **k** The zoom view of **j**. l Learning-forgetting-relearning process over seven cycles. **m** PPF index variation with the time interval (Δt) of photonic pulse pairs. Inset: PSC under blue light (intensity: 9.5 mW cm^−2^, duration: 1 s) pulse pairs with a 10 s time interval.

To enhance the capabilities of optoelectronic synapses and enable them to adapt to diverse shapes and configurations for a range of applications, such as wearables, it is essential for the devices to exhibit robust stability even when subjected to bending conditions. Figure 5a–c shows the photograph of the flexible device during optical testing. To evaluate the photonic synaptic features, the device was bent from 4 to 2 cm. The uniformity and stability of the devices under bending conditions were assessed by performing general voltage sweeps under both flat and bending (2 cm) conditions, as depicted in Fig. 5d. To facilitate the assessment of cyclic stability, 100 DC cycles of *I–V* sweeps were recorded, and the RS parameters were depicted in Fig. 5e. Under bending conditions, both the RS characteristics and the SET/RESET variabilities, as well as the memory windows of the devices, were observed to be similar to those under flat conditions. This indicates that the flexible optoelectronic synapses maintain consistent performance and reliability even when subjected to mechanical deformation, demonstrating their potential for use in flexible and wearable electronic applications. The device-to-device uniformity of the 20 randomly selected device show excellent stability in both LRS and HRS states with a bending of 2 cm (Fig. 5f).

Figure 5g depicts the PSC response of the device in flat (4 cm) and bent (2 cm) condition when exposed to optical light (456 nm) at 9.5 mW cm^−2^ for 5 s. The PSC of the device was increased for both flat and bent devices. After removing the optical light, both devices (flat and bent) exhibit a gradual decay in PSC instead of quickly returning to the initial current. The PSC of the device exhibits modulation when subjected to varying light intensities and time duration, as depicted in Fig. 5h and i, respectively. A higher intensity of light and higher time duration corresponds to an augmented current conductance, and the rate of its decay is also influenced by the intensity of light and time duration. In contrast, lower intensity light and lower time results in a slight increase in current conduction, which rapidly reverts to its initial state upon power interruption, akin to STM processes observed in the human brain. Conversely, with the removal of higher intensity light and higher time duration, the PSC exhibits a gradual decay and maintains an elevated level above its initial state, like the concept of LTM in the human brain. In conclusion, the optoelectronic memristive device demonstrates the capability to transform its PSC response from STM to LTM by manipulating either the intensity of light or the time duration of exposure.

Through the continuous cycling of light on and off, PSC response of the device exhibits a notable enhancement with an increasing number of light pulses, as depicted in Fig. 5j. The PSC of the device escalates from 57 to 83 nA following 30 successive on/off cycles. Figure 5k shows the zooming part of Fig. 5j. Furthermore, we have demonstrated the learning-forgetting process for our flexible device by repeatedly switching the light on and off, as illustrated in Fig. 5l. Illuminating the device corresponds to learning and relearning tendencies, whereas extinguishing the light signifies the act of forgetting. The PSC of the device exhibits an increment with light exposure, followed by a decline to an intermediary level after a certain duration, indicating a gradual fading of acquired knowledge over time. Through the persistent repetition of the learning or relearning cycle, the device's PSC experiences incremental enhancements (A_6_ > A_5_ > A_4_ > A_3_ > A_2_ > A_1_), underscoring the potential of previously assimilated information to significantly expand memory capacity. Ultimately, the device attains its peak PSC level (A_6_) after six consecutive learning and relearning phases, representing the successful transition from STM to LTM within the optoelectronic memristive device. We further investigate the PPF of the flexible device, as shown in Fig. 5m. The PPF was calculated using the same equation as electrical PPF, as shown in Fig. 4e. The curve demonstrates excellent mimicry of synaptic photonic PPF features, which is an essential synaptic function in neuromorphic vision systems. These excellent features in the flexible device make it highly efficient in wearable applications.

### Optoelectronic Image Mapping

**Fig. 6:**
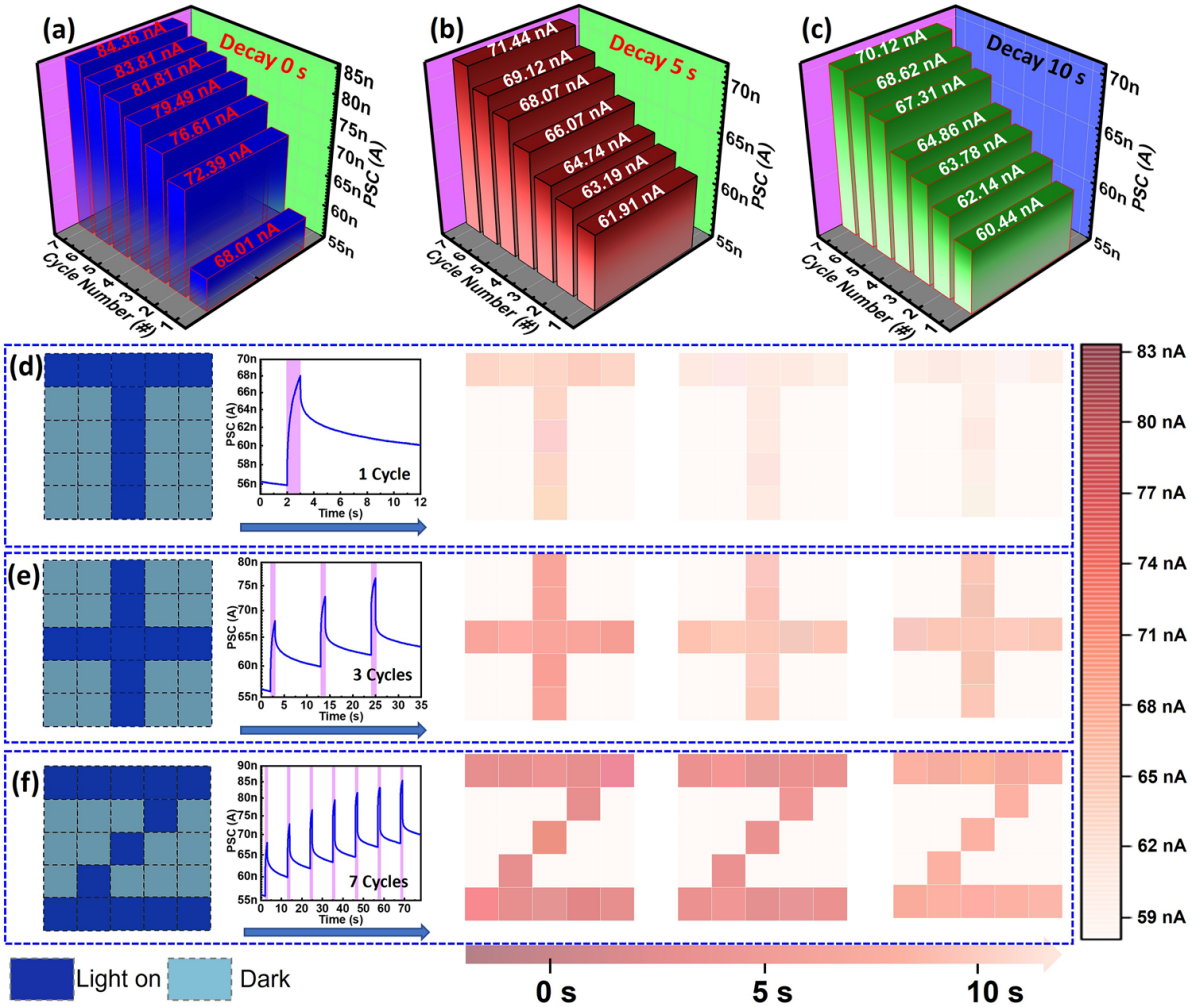
The current conductance of the optoelectronic memristor after various number of cycles at **a** 0 s, **b** 10 s, and **c** 5 s decay time. The image mapping for current conductance after **d** 1 cycle, **e** 3 cycles, and **f** 7 cycles

Observations of visual memory suggest that memory retention can be improved by increasing the number of cycles or by extending the stimulus duration, also known as memory recall. In our study, we evaluated the visual memory function using a 5 × 5 device array, which follows the "learning-forgetting-relearning" process. Similar to the human visual system, this array has the ability to detect optical vision pulse and produce the corresponding PSC change. Figure 6a displays the photocurrent response for varying numbers of cycles (1, 2, 3, … 7). As anticipated, the PSC response rises with more cycles, with a 16 nA difference between the 1st and 7th cycles. Figure 6b, c show the conductance state for different stimulus numbers at decay times of 5 and 10 s, respectively. The findings disclose that memory strength increases with a higher number of stimuli, which leads to longer decay times for forgetting. To assess the sensing ability of our visual perception, we introduced the characters "T," " + ," and "Z" with 1, 3, and 7 cycles, respectively (Fig. 6d–f). We observed that the letter "T" can only be remembered briefly and becomes blurred over time until it's forgotten, indicating that a single cycle is not sufficient for retaining image information. In contrast, the letter "Z" for 5 cycles presented a clear image contour even after a decay time of 10 s. By adjusting the light stimulus, we were able to efficiently modulate the device conductance following the "learning-forgetting-relearning" rules without requiring any additional voltage pulse, thus effectively emulating the human visual perception property. These results demonstrate the array's ability to learn and simulate visual functions and highlight its potential as an artificial visual perception in future electronic vision systems. We have demonstrated our device for human visual system by varying the light intensity and fixed illumination time in Note-S8.

### ECG Signal Visualization

**Fig. 7:**
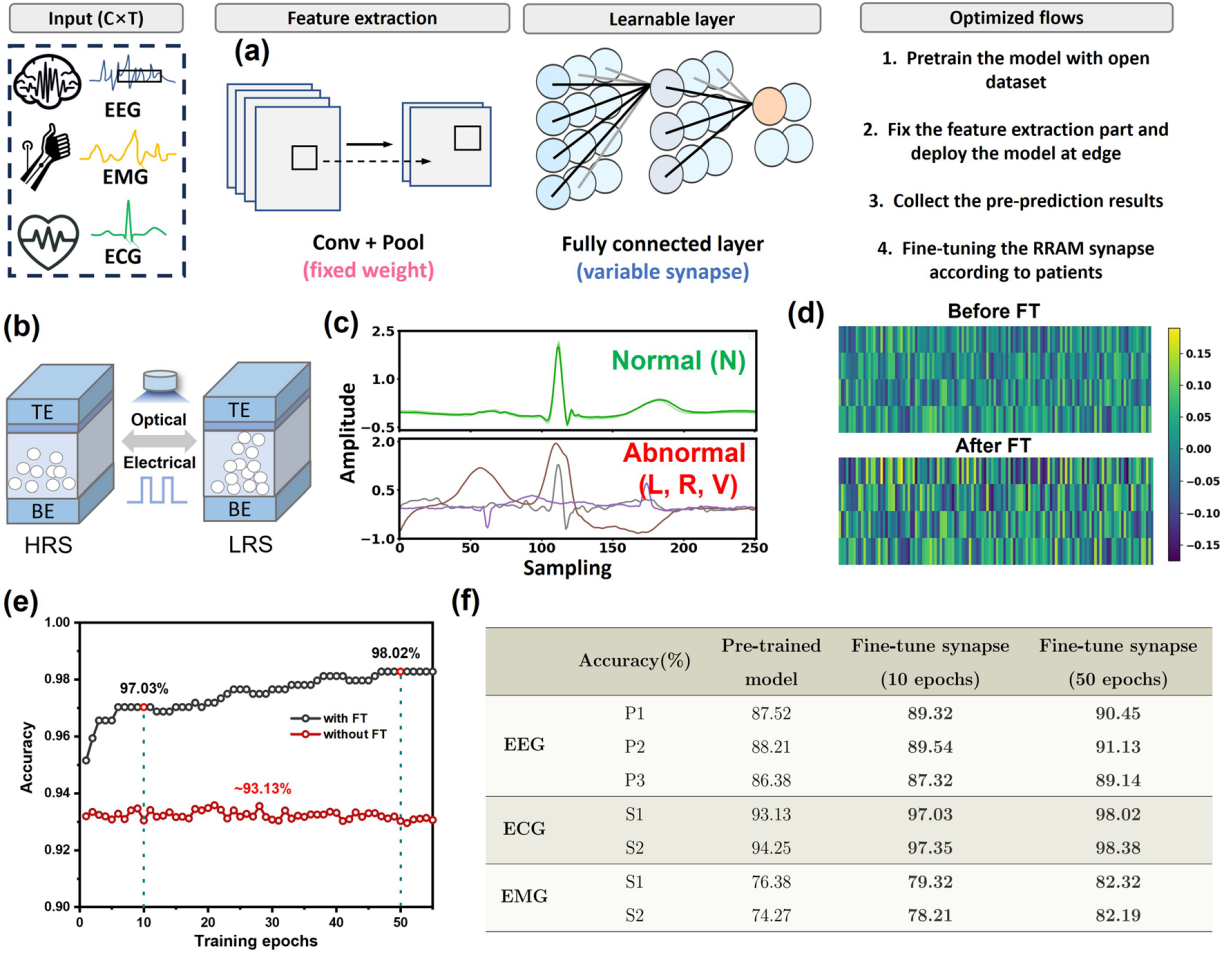
**a** Concept of edge biomedical AI processor. **b** Conductance state change on ITO/ZnO/HfO_x_/Pt crossbar array. **c** ECG signal visualization for normal and abnormal events. **d** Weight map visualization before and after fine-tune process. **e** Training accuracy behaves with and without fine-tune process for ECG based arrhythmia detection. **f** Comparison table of training accuracy for three bio-signals

Figure 7a shows the process flow of our wearable biomedical processing system. First, the network is pretrained on three open-source datasets for three biomedical tasks (see Note-S9). Network details are available in supplementary information Table S2. For feature extraction part, we fixed the weights of convolutional layer and pooling layer to extract general feature. For the fully connect layer, the weights are initially established through the pre-training process, but subsequently fine-tune based on specific patient or new subjects. The optimization fine-tune process can be found in Note-S10. Figure 7b shows the conductance state change in our hardware synapse with both electrical and optical programming techniques. Figure 7c presents the ECG signal visualization for normal and abnormal events, which can be classified by our network model. Figure 7d illustrates the weight state change before and after fine-tune process. The fine-tune process adjusts network weight to have a better performance under new subject or patient. With the ability of programmability and flexibility, our memristor device can serve as hardware synapse and implementation of synaptic weight in neural networks. In ECG-based arrhythmia detection, as new subjects are introduced, the fine-tuned model yields better performance. Specifically, it leads to a classification accuracy of 97.03% after 10 epochs and 98.02% after 50 epochs, as illustrated in Fig. 7e. In contrast, the original model maintains a stable accuracy of approximately 93.13%. In Fig. 7f, we summarize the training result for three biosignals in the table. These results clearly demonstrate that the fine-tuned model with memristor exhibits significantly improved performance compared to the original configuration.

Integrating this technology into wearable or implantable devices, one limitation arises in terms of the complexity involved in integrating functionalities, particularly in circuit design, to balance speed and precision in optosynaptic operations like EPSC for more accurate biological synapse emulation. Resolving noise and interference issues, especially in dense memristor arrays with optical stimulation, becomes crucial for robust and reliable operation. Future directions will focus on developing advanced circuit designs, including neuromorphic architectures and on-chip photosynapse, to optimize optosynapse-memristor systems for specific applications. This involves integrating machine learning algorithms for adaptive control and optimization of optosynaptic networks, enabling real-time learning and adaptation. Additionally, exploring interfaces with biological systems, such as brain-inspired interfaces, holds promise for applications in neuroprosthetics, brain-machine interfaces, and neural computing. Overcoming scalability and manufacture challenges is crucial to realizing large-scale optosynaptic memristor arrays for practical applications in artificial intelligence and computing.

## Conclusion

In this study, a back-end-of-line compatible optoelectronic flexible memristor is designed for both physiological signals processing and visual processing applications. The crossbar array memristive device in combination with a transfer learning approach for healthcare applications. These applications encompass EEG-based seizure prediction, EMG-based gesture recognition, and ECG-based arrhythmia detection. Through experimentation on three distinct biomedical datasets, we note a significant enhancement in classification accuracy for the pretrained model, specifically 2.93% improvement for EEG, 4.90% for ECG, and 7.92% for EMG, respectively. The device demonstrates advanced synaptic properties, including long-term and short-term memory, as well as learning, forgetting, and relearning when exposed to visible light when device was bent to 2 cm. These advanced properties can enhance information processing capabilities for neuromorphic applications. Notably, STM can be transformed into LTM by adjusting the light intensity and duration of exposure. In order to exploit the device's light-responsive features, a 5 × 5 synaptic array was created to showcase its potential use in artificial visual perception. The combination of these features in a single memristive cell makes it highly suitable for applications in image recognition and artificial visual perception for wearable applications.

## Supplementary Information

Below is the link to the electronic supplementary material.

Supplementary file1 (DOCX 2904 KB)
