# Correction: Highly Efficient Back-End-of-Line Compatible Flexible Si-Based Optical Memristive Crossbar Array for Edge Neuromorphic Physiological Signal Processing and Bionic Machine Vision

**DOI:** 10.1007/s40820-024-01490-6

**Published:** 2024-08-08

**Authors:** Dayanand Kumar, Hanrui Li, Dhananjay D. Kumbhar, Manoj Kumar Rajbhar, Uttam Kumar Das, Abdul Momin Syed, Georgian Melinte, Nazek El-Atab

**Affiliations:** Smart, Advanced Memory Devices and Applications (SAMA) Laboratory, Electrical and Computer Engineering, Computer Electrical Mathematical Science and Engineering, King Abdullah University of Science and Technology (KAUST), 23955-6900 Thuwal, Saudi Arabia

**Correction to: Nano-Micro Lett. (2024) 16:238** 10.1007/s40820-024-01456-8

Following publication of the original article [1], the authors noticed a mistake in the Supplementary file, more specifically in figures S11 and S12 where they used by mistake the same sub-figures.

The original article [1] has been corrected.
