# Supplementary material for: Highly Efficient Back-End-of-Line Compatible Flexible Si-Based Optical Memristive Crossbar Array for Edge Neuromorphic Physiological Signal Processing and Bionic Machine Vision
Source: Nanomicro Lett. 2024 Jul 8;16:238. doi: 10.1007/s40820-024-01456-8 (PMC11231128; doi:10.1007/s40820-024-01456-8)
Supplement: Supplementary file 1 — Supplementary file1 (DOCX 2904 KB) [file 40820_2024_1456_MOESM1_ESM.docx]

Supporting Information for

**Highly Efficient Back-End-of-Line Compatible Flexible Si-Based Optical Memristive Crossbar Array for Edge Neuromorphic Physiological Signal Processing and Bionic Machine Vision**

Dayanand Kumar^1,^ǂ, Hanrui Li^1,^ǂ, Dhananjay D. Kumbhar^1^, Manoj Kumar Rajbhar^1^, Uttam Kumar Das^1^, Abdul Momin Syed^1^, Georgian Melinte^1^, and Nazek El-Atab^1,^*

^1^Smart, Advanced Memory Devices and Applications (SAMA) Laboratory, Electrical and Computer Engineering, Computer Electrical Mathematical Science and Engineering, King Abdullah University of Science and Technology (KAUST), Thuwal 23955-6900, Saudi Arabia

*ǂ* Dayanand Kumar and Hanrui Li have contributed equally to this work.

*Corresponding author. E-mail: [nazek.elatab@kaust.edu.sa](mailto:nazek.elatab@kaust.edu.sa) (Nazek El-Atab)

**Note-S1 Comprehensive Comparison of Previously Reported HfO_x_ and ZnO-based Devices with the Current Work**

**Table S1** shows a compre ensive comparison between previously reported oxides based memristive synapses and this work

**
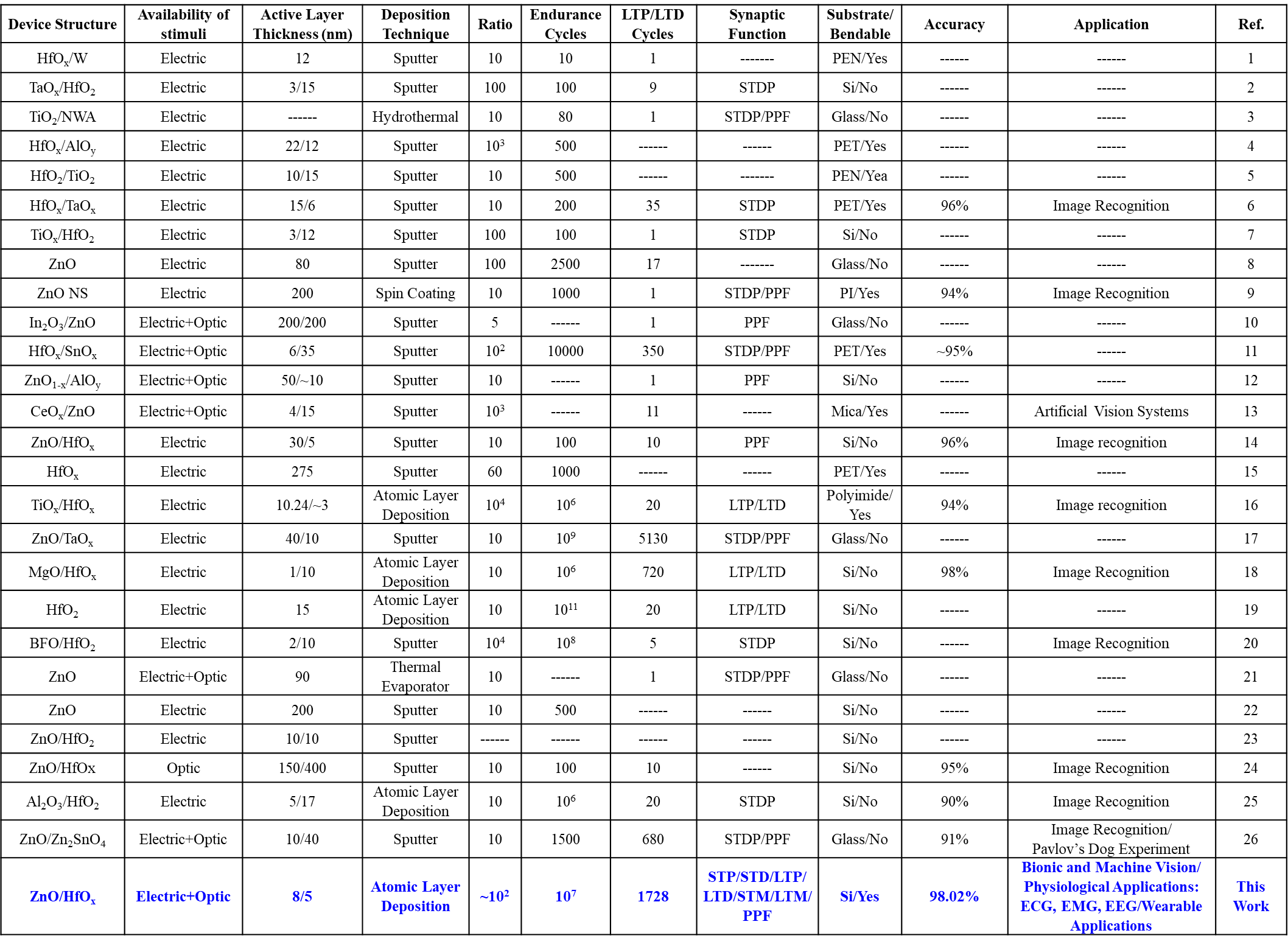
**

**Note-S2 Crossbar Array Fabrication Procedure**


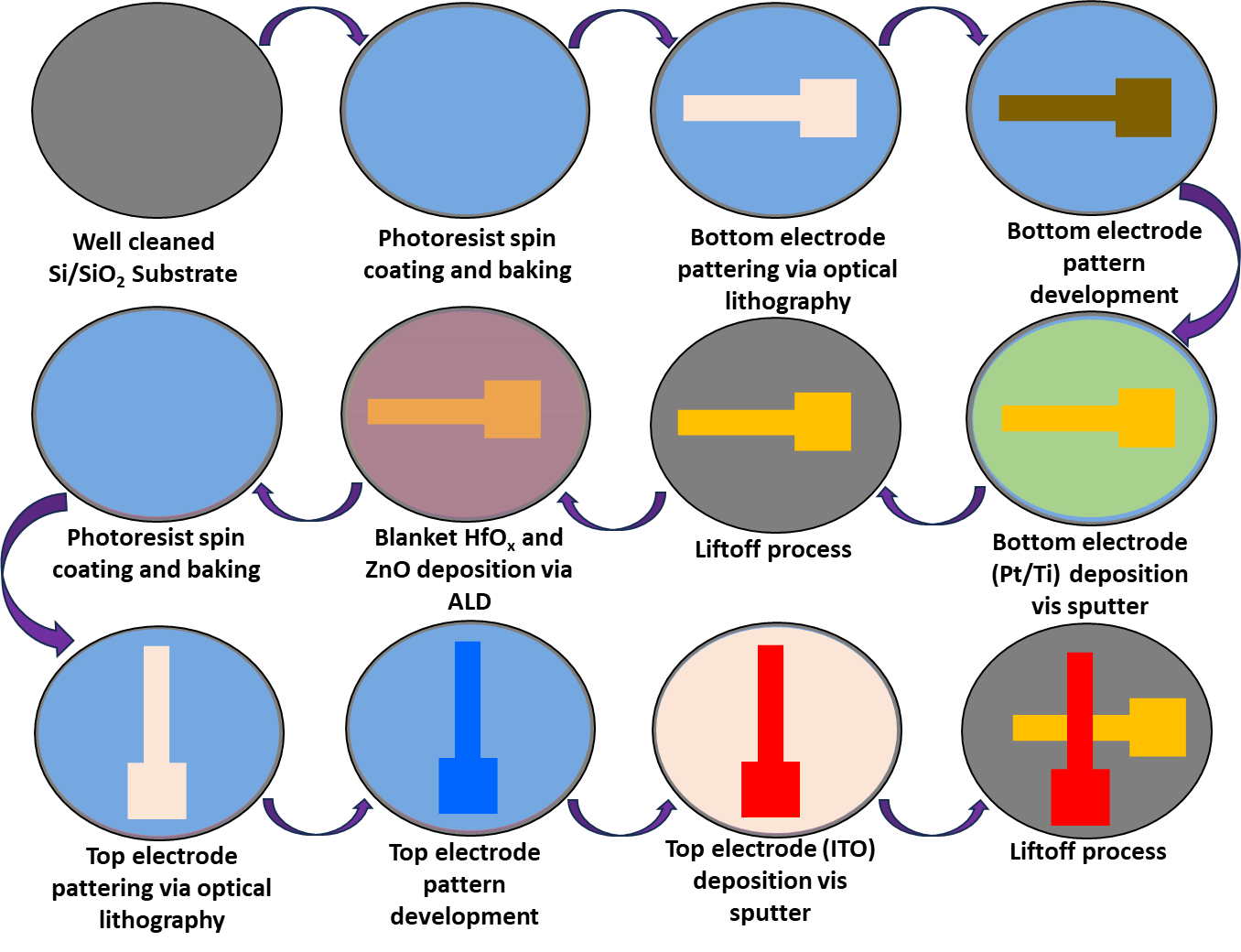


**
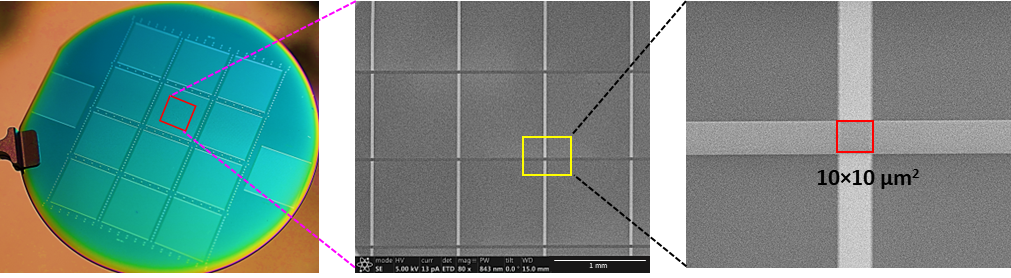
**

**Fig. S1** Fabrication procedure of the crossbar array on 4-inch Si wafer, optical photograph of the integrated crossbar array and zoom in high-resolution SEM image of crossbar array and zoom in SEM image of single crossbar cell

**Note-S3 Forming Process of the ITO/ZnO/HfO_x_/Pt Device**

A forming procedure with controlled current compliance is mandatory for the fresh device to initiate the resistive switching characteristics and to protect the device from permanent dielectric breakdown. **Fig. S2** shows the typical forming behaviors of the ITO/ZnO/HfO_x_/ITO device, where the forming process with a current compliance of 200 µA was occurred at forming voltages of about 2.5 V.

**Fig. S2** shows the forming process of the ITO/HfO_x_/ZnO/Pt device

**Note-S4 Conduction Mechanism of the Device Based on Oxygen Vacancies**

To understand the conduction mechanism in the ITO/ZnO/HfO_x_/ITO memristor, O 1s spectra were recorded for HfO_x_ and ZnO thin films, which are described in **Fig. S3** **a** and **S3** **b**, respectively. To obtain the oxygen vacancies concentration in the HfO_x_ and ZnO layers, X-ray photoelectron


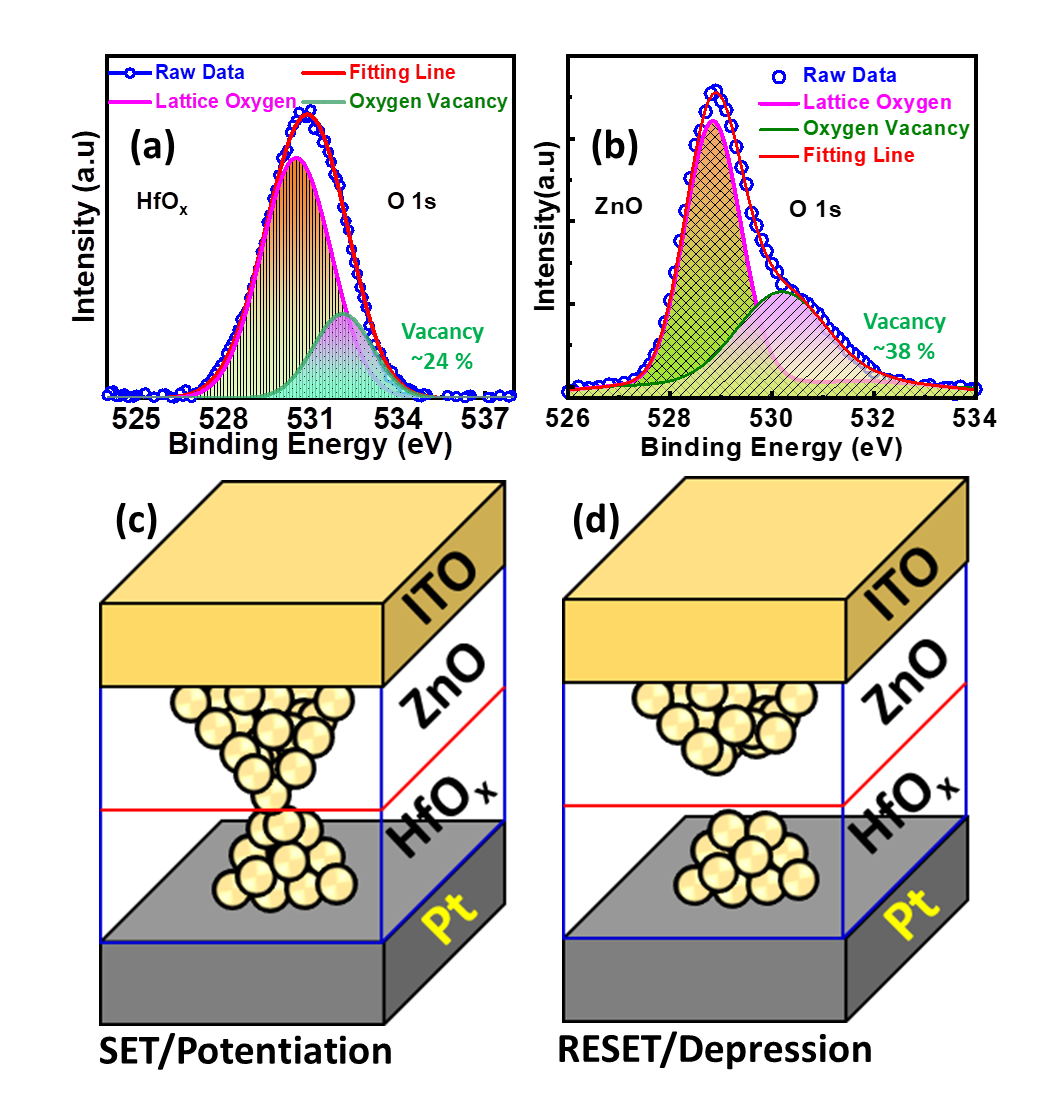


**Fig. S3** **a,** **b** show the XPS spectra of HfOx and ZnO, respectively. **c,** **d** show the CF model in and RESET condition spectroscopy (XPS) was observed.

The X-ray’s beam size was set to ∼0.25 mm^2^. The profile of the O 1s core level spectra was done by successive etching of the sample for ∼5 s to remove the upper surface layer. The core level O 1s spectra of HfO_x_ and ZnO layers were fitted using Gaussian functions method. The O 1s spectrum of HfO_x_ and ZnO layers can be deconvoluted into two Gaussian peaks. The major peaks which are located at the binding energies of 530.4 and 532.3 eV represent the lattice oxygen, whereas minor peaks which are situated at 528.5 and 530.2 eV are ascribed to the oxygen vacancies in the HfO_x_ and ZnO layers, respectively. From the XPS analysis, we found that the oxygen vacancies concentration in HfO_x_ and ZnO are about 24 and 38 %, respectively. The oxygen vacancies perform a vital role to form the conducting filament (CF) in the HfOx and ZnO layers. Based on the concentration of oxygen vacancies in both HfO_x_ and ZnO layers (**Fig. S3a**, **b**), we confirm the CF model in our device, as shown in **Fig. S3c**, **d**. Previous reports have proven that different amounts of oxygen vacancies create the hourglass shape CF in the bilayer devices [S27, S28]. When we applied the positive voltage on the ITO TE, oxygen vacancies are generated in the HfO_x_ layer and move toward the Pt BE to form the conical shape filament in the HfO_x_ layer. As the oxygen vacancies are enhanced in the HfO_x_ layer, HfO_x_ becomes electrically conductive and plays as a “virtual electrode” [S27, S28]. It is well known that the conductive virtual electrode, which is part of the CF, also consists of oxygen vacancies. Now, the “virtual electrode” as in the HfO_x_ thin film tends to be the seed for CF regrowth in the ZnO layer. Based on the resistive switching phenomenon, the growth and broken of the CF happens at the ZnO/HfO_x_ interface. When the device is switched to HRS, the root of the CF does not dissolve completely during the RESET process. Therefore, In the ITO/ZnO/HfO_x_/Pt device, the CF is preferably connected or breached at the ZnO/HfOx interface, and the HfOx layer behaves as a virtual electrode. This resistive switching mechanism is believed to improve the neuromorphic characteristics of the ITO/ZnO/HfOx/Pt device. Therefore, the formation and rupture of the CF happen at ZnO/HfOx interface. This hourglass-shaped CF in ZnO/HfOx bilayer enhances the performance of the bilayer device [S29, S30].

**Note-S5 Electrical Characteristics of the ITO/ZnO/HfOx/Pt Device**


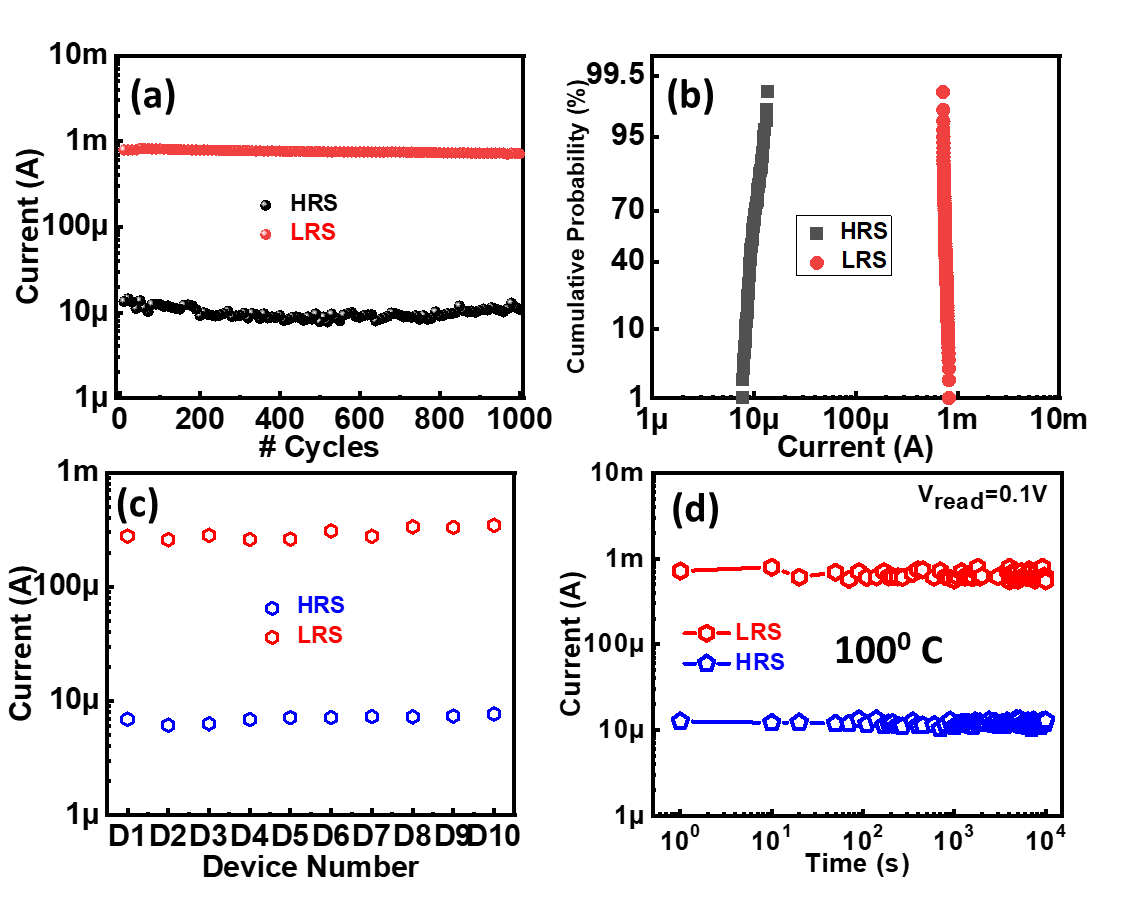


**Fig. S4** **a** DC endurance. **b** Cycle to cycle uniformity. **c** Device to device stability. **d** High temperature retention test

The DC endurance of the device is shown in **Fig. S4a**. The device demonstrates excellent stability in both low resistance state (LRS) and high resistance state (HRS) for more than 1000 DC cycles without any humiliation. To confirm the excellent stability of the ITO/ZnO/HfO_x_/Pt memristive device, the cycle-to-cycle consistency was measured, which is illustrated in **Figure S4b**. The cycle to cycle of the device was measured using 100 continuous cycles. The device indicates high consistency in both LRS and HRS without any disruption. The device-to-device uniformity of the device shows the excellent stability in both LRS and HRS states (**Fig. S4c**). The retention test of the device was also measured, which is shown in **Fig. S4d**. The device confirms the stable retention at high temperature (100^0^ C) for both high resistance state (HRS) and low resistance state (LRS).

**Variability and reliability of fabricated devices**

**Cyclic Variability:** The stability assessment involved analyzing 100 consecutive sweeping I-V curves, depicted in **Fig. S5a**. These results highlight the robust cycle stability and repeatability of the device, evidenced by the consistent behavior of the I-V curves across increasing sweeping cycles. Furthermore, **Fig. S5b, c** showcase the endurance performance over 100 cycles, focusing on the extracted parameters V_SET_, I_SET_, V_RESET_, and I_RESET_ from the IV characteristics. The proposed memristor demonstrates reliable reproducibility, maintaining acceptable variability compared to previous reports [S31-S34]. Statistical analyses of the set and reset voltages, along with their respective currents, are presented in **Fig. S6**, where histograms display the distributions fitted using the Normal distribution. The statistical count accurately represents V_SET_ and V_RESET_ within acceptable ranges. The analysis reveals a concentrated distribution for V_SET_ around 0.76 V and for V_RESET_ around -0.51 V, with I_SET_ and I_RESET_ values around 35.7 µA and -3.13 mA, respectively, indicating a stable operational range. The standard deviations (σ) for V_SET_ and V_RESET_ voltages are measured at 0.214 V and -0.078 V, respectively, while those for I_SET_ and I_RESET_ are 21.4×10^-6^ and 3.134×10^-4^, respectively. This homogeneity in operation voltage establishes a strong foundation for the practical application of the memristor device, ensuring consistent and reliable performance [S31-S34]. Additionally, **Fig. S7** illustrates the cycle-to-cycle cumulative distribution function (CDF) of switching voltages for the same data. It shows consistent variability across each particular condition, further confirming the stability and reliability of the device under varying operational cycles [S31-S35].


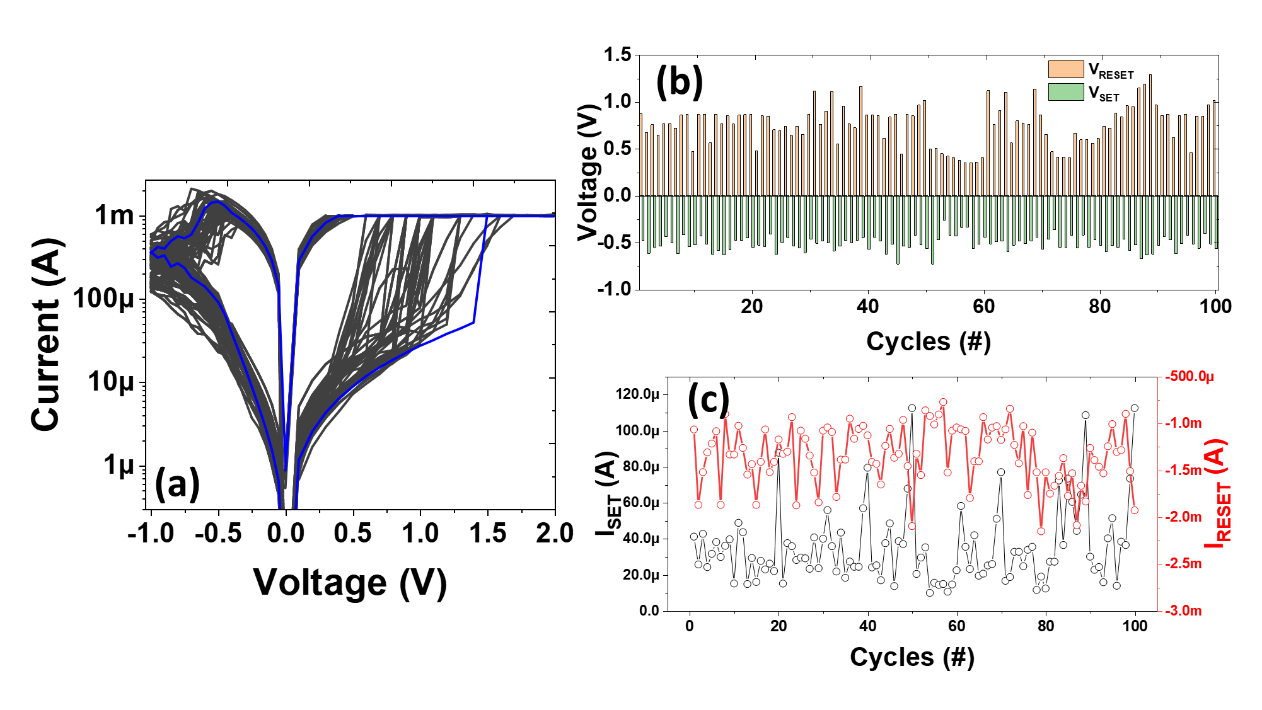


**Fig. S5 (a)** The stability assessment involved analyzing 100 consecutive sweeping I-V curves. **(b)** and **(c)** showcase the endurance performance over 100 cycles, focusing on the extracted parameters V_SET_, I_SET_, V_RESET_, and I_RESET_ from the IV characteristics


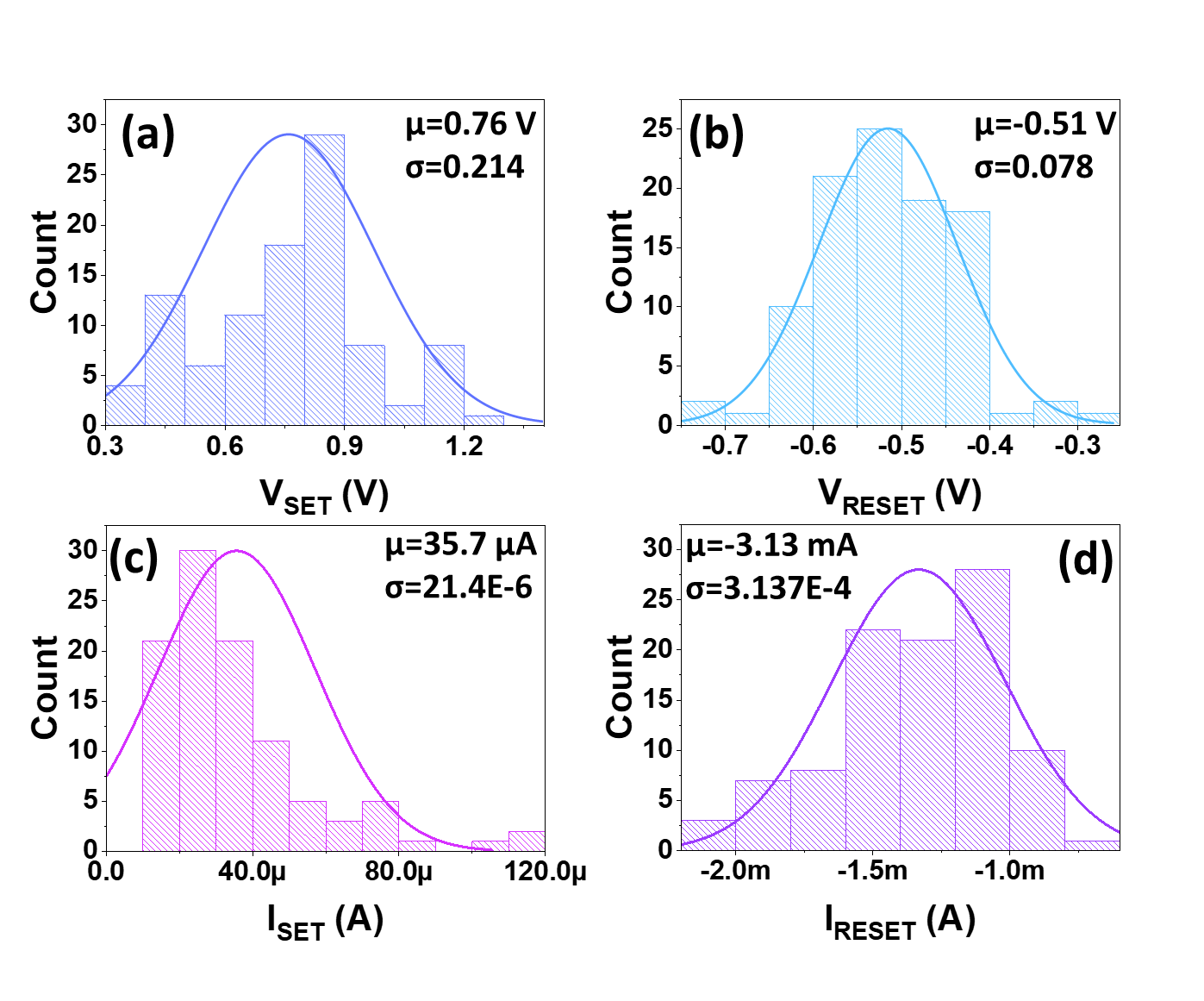


**Fig. S6** The histograms display the distributions fitted using the Normal distribution statistical count accurately represents **(a)** V_SET_ and **(b)** V_RESET_, **(c)**I_SET_ and **(d)** I_RESET_


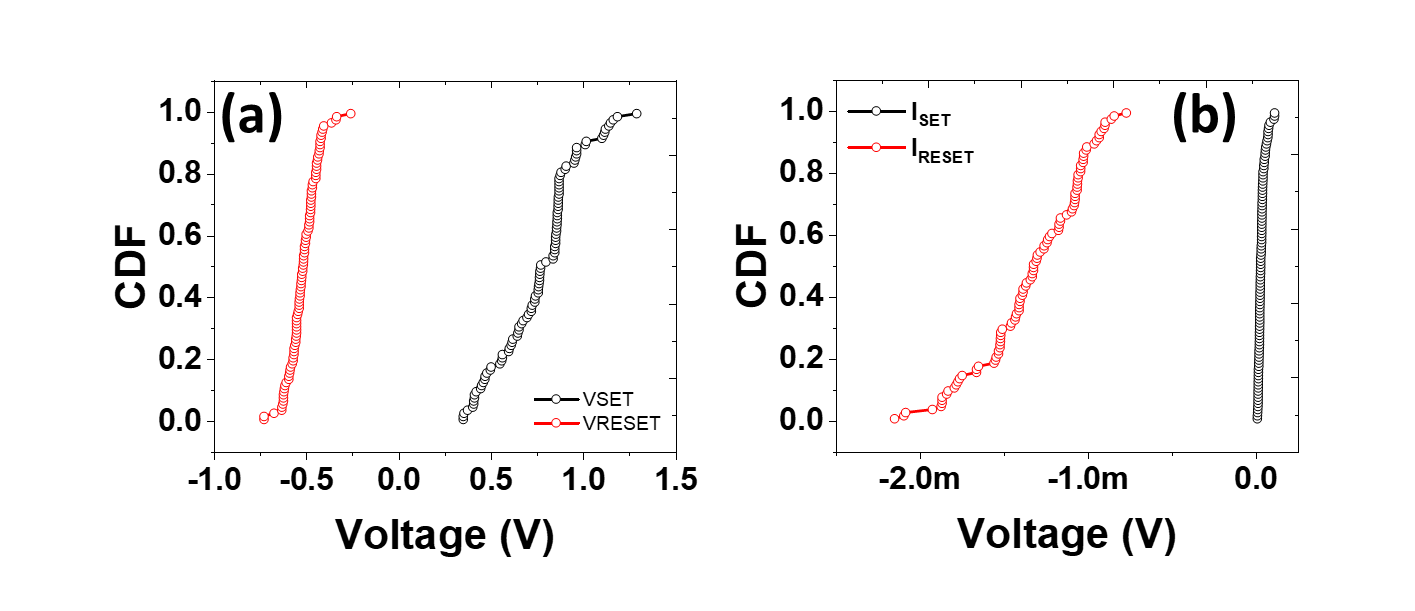


**Fig. S7** Cumulative distribution function (CDF) on **(a)** switching voltages and **(b)** switching currents

**Device variability:** The fabricated crossbar array size of (5×5) with 14 batches shows a device production yield of ~92.00% (an average of 23 devices are in a functional condition out of a total of 25 on each array) while ~8% devices are in non-functional condition as depicted in **Fig. S8**. **Figs. S9** and **S10** present a comprehensive analysis of the resistive switching (RS) behavior observed across an array of 22 devices. The evaluation encompassed double-sweep voltage ramps ranging from 0 to 2 V for the set process and from 0 to -1 V for the reset process. This thorough assessment aimed to evaluate the uniformity of the RS phenomenon across D2D and C2C variations. The RS characteristics depicted in **Fig. S9** were derived from all selected working devices on array, contributing to a nuanced understanding of RS stability and uniformity under diverse operational conditions [S31-S34]. The rigorous analysis showcased in **Fig. S9** reveals that all devices in the array exhibit exceptional reliability in their RS characteristics. This finding underscores the robustness of the device's performance across the array. Furthermore, such representations are recommended for D2D variability studies, as a comprehensive analysis necessitates showcasing results across a wide array of devices [S31-S35]. To further elucidate the variability in RS performance, exploring box plots is warranted. These plots can effectively depict both C2C and D2D variability across the array. The analysis of 10 cycles per device, as shown in **Fig.** **S10a-d**, demonstrates acceptable C2C variability across all devices. This observation highlights the robustness and consistency of RS performance, showcasing the promising stability of these devices under varying operational conditions. The data presented in **Figs. S9** and **S10a-d** provide valuable insights into the uniformity, reproducibility, and stability of RS characteristics in the fabricated array of devices. This information is crucial for understanding and optimizing the performance of these devices for practical applications.

Similarly, to comprehensively assess the reliability and reproducibility of devices across multiple fabricated arrays on the wafer, a set of randomly selected devices underwent rigorous testing through repetitive I-V cycles. The results from 10 I-V cycles of these randomly selected devices are presented in **Figs. S11** and **S12**, providing detailed insights into the device's performance. These findings demonstrate exceptionally good agreement and reproducibility across C2C, D2D, and batch-to-batch variations. The consistent performance observed throughout these variations underscores the robustness and reliability of the devices under diverse operational conditions [31-34]. **Figs. S11** and **S12** illustrate the uniformity and stability of key parameters across multiple cycles and devices, further reinforcing the high level of reproducibility achieved in the fabrication process. This level of agreement is crucial for ensuring the consistency and predictability of device behavior, laying a solid foundation for their practical application in various contexts [S31-S35].


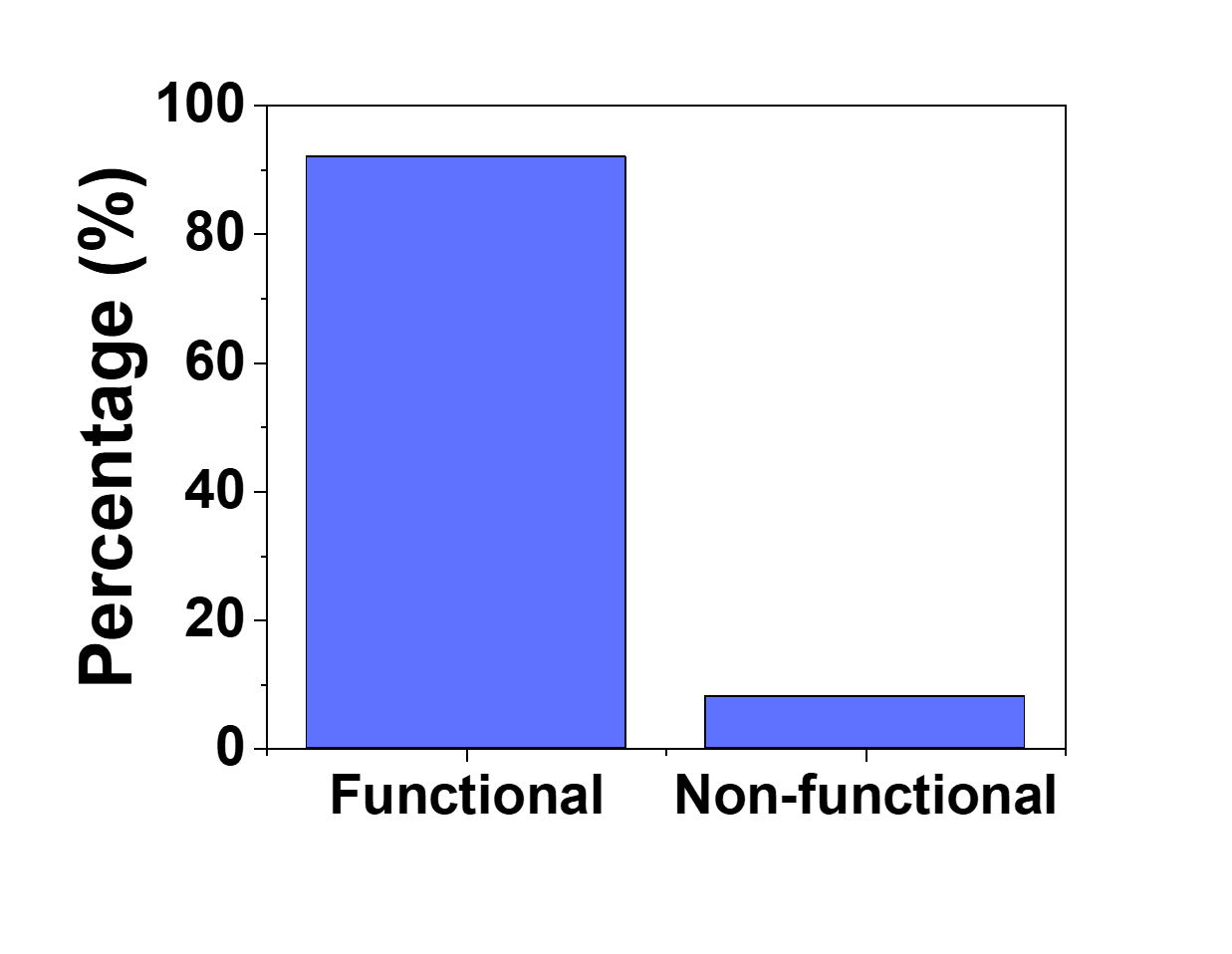


**Fig. S8** average percentage of functional and non-functional devices across the fabricated crossbar array


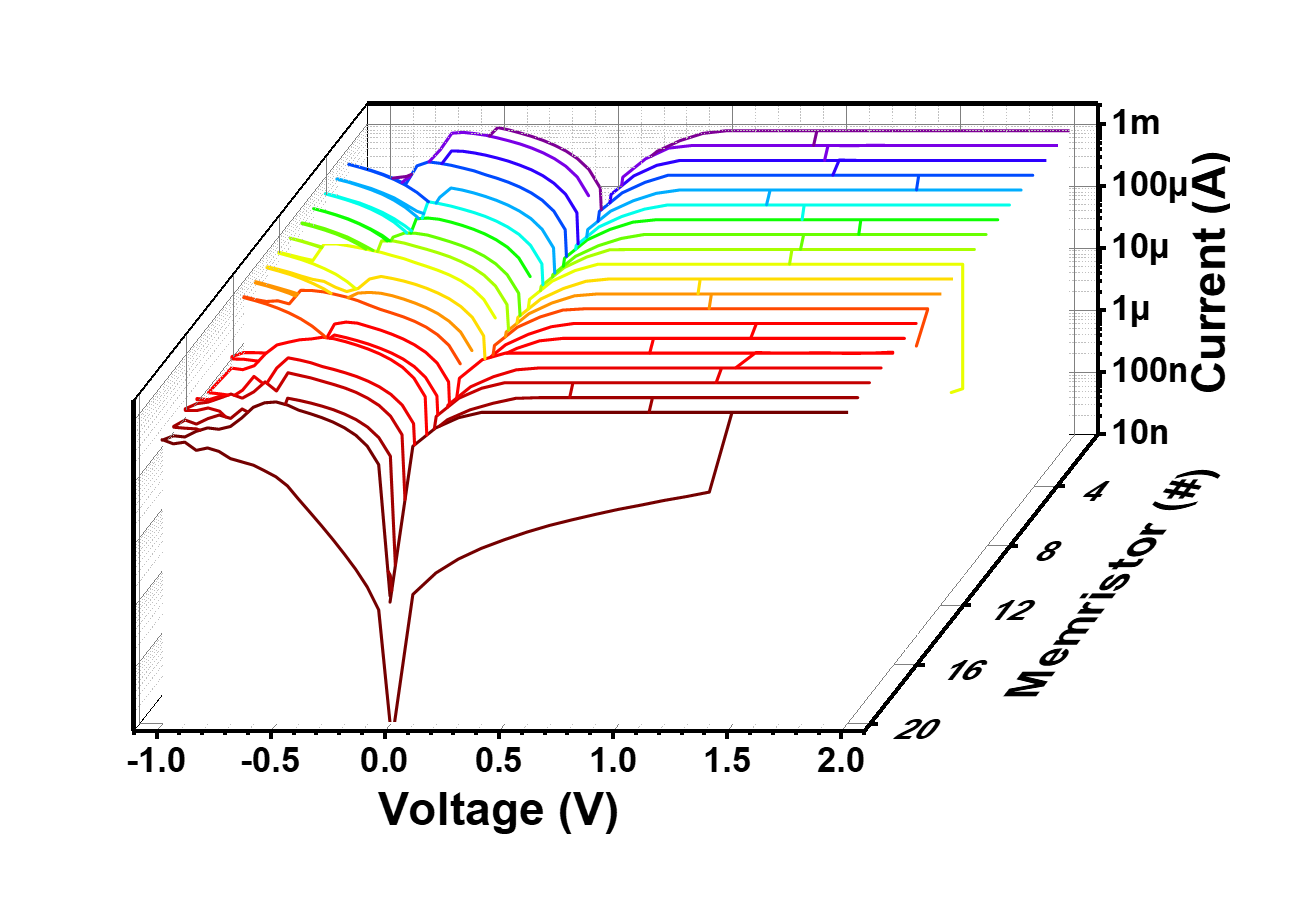


**Fig. S9** Device variability of multiple devices through *IV* curves across the array


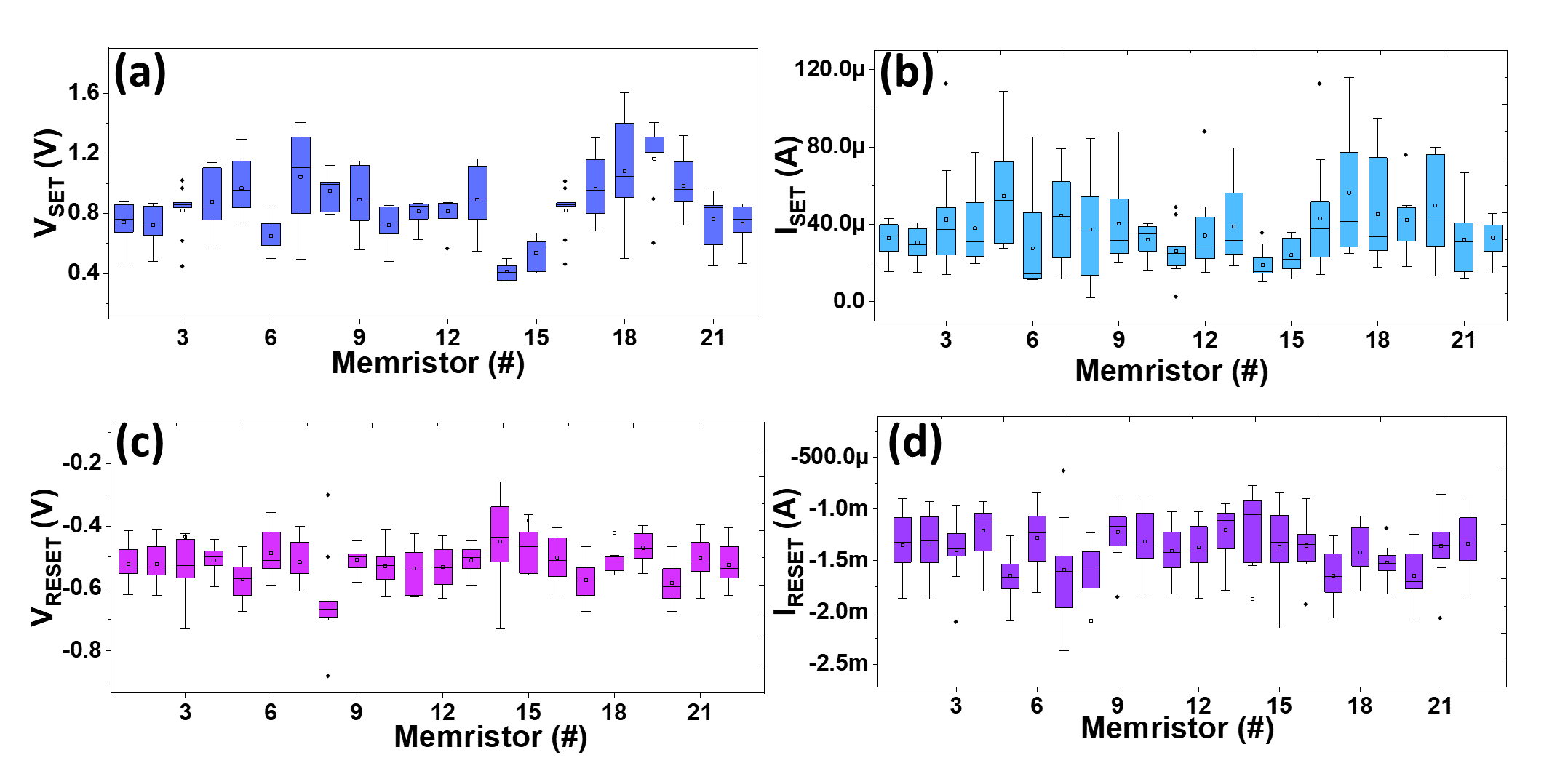


**Fig. S10** Devices variability across key RS parameters **a** V_SET_, **b** I_SET_, **c** V_RESET_, and **d** I_RESET_


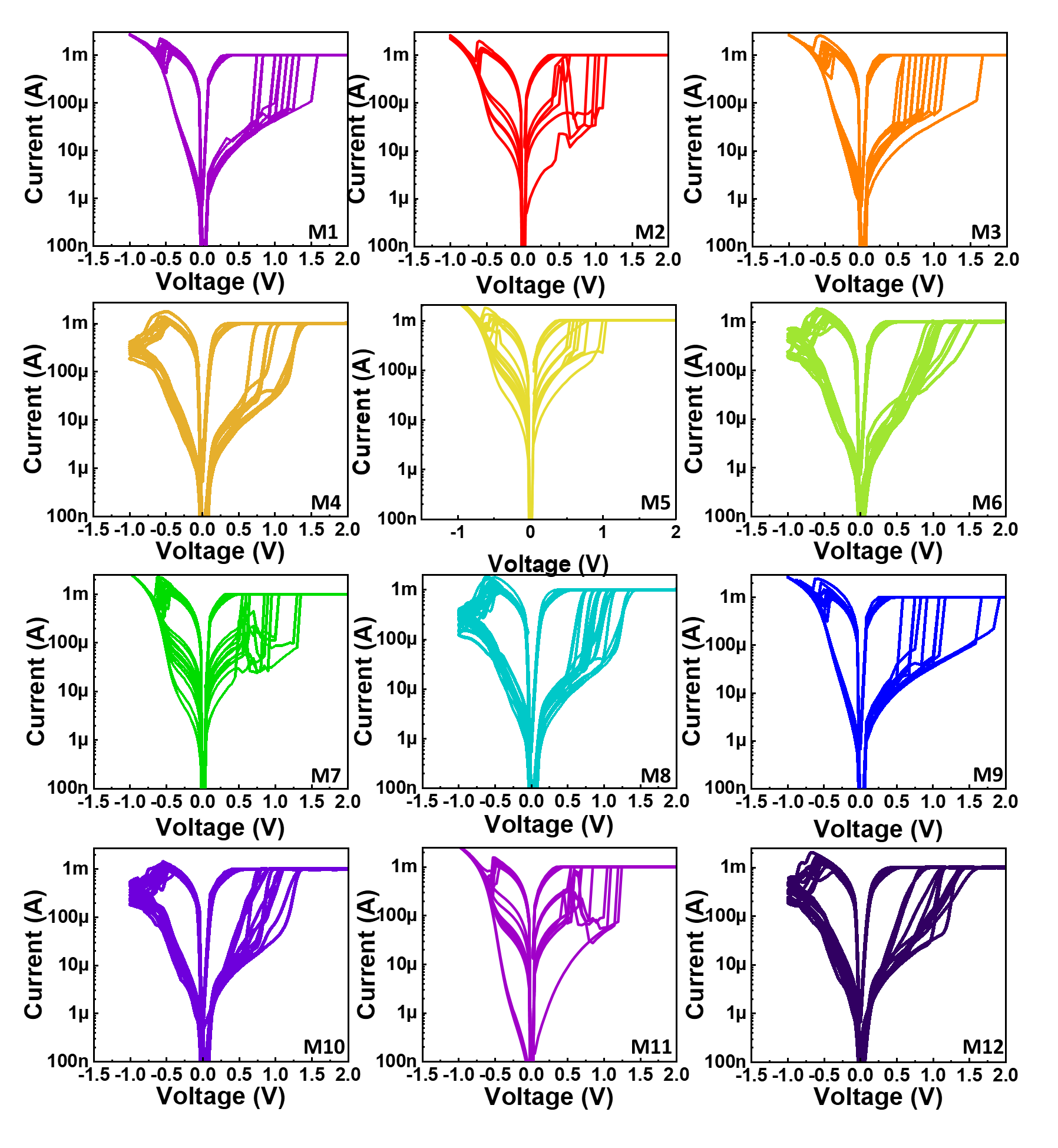


**Fig. S11** Cyclic variability in 10 cyclic IV sweeps across randomly chosen 28 devices [M1-M12]


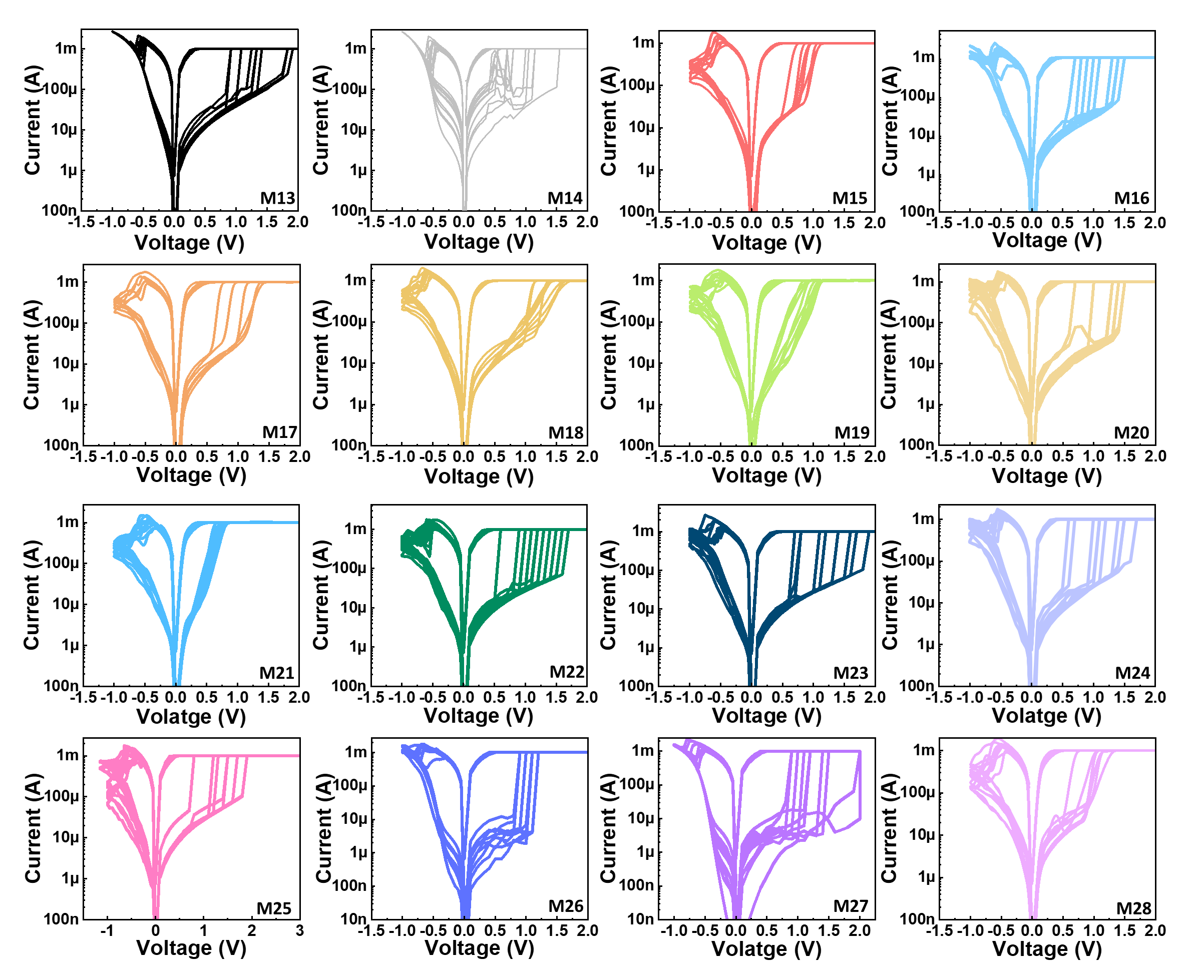


**Fig. S12** Cyclic variability in 10 cyclic IV sweeps across randomly chosen 28 devices [M13-M28].

**Note-S6 Read Voltage Dependent Study of the Device**

**Fig. S13** read voltage-dependent responses photo synapses from 10 to 30 mV

**Note-S7 Mechanism for Photonic ITO/ZnO/HfO_x_/Pt Device**


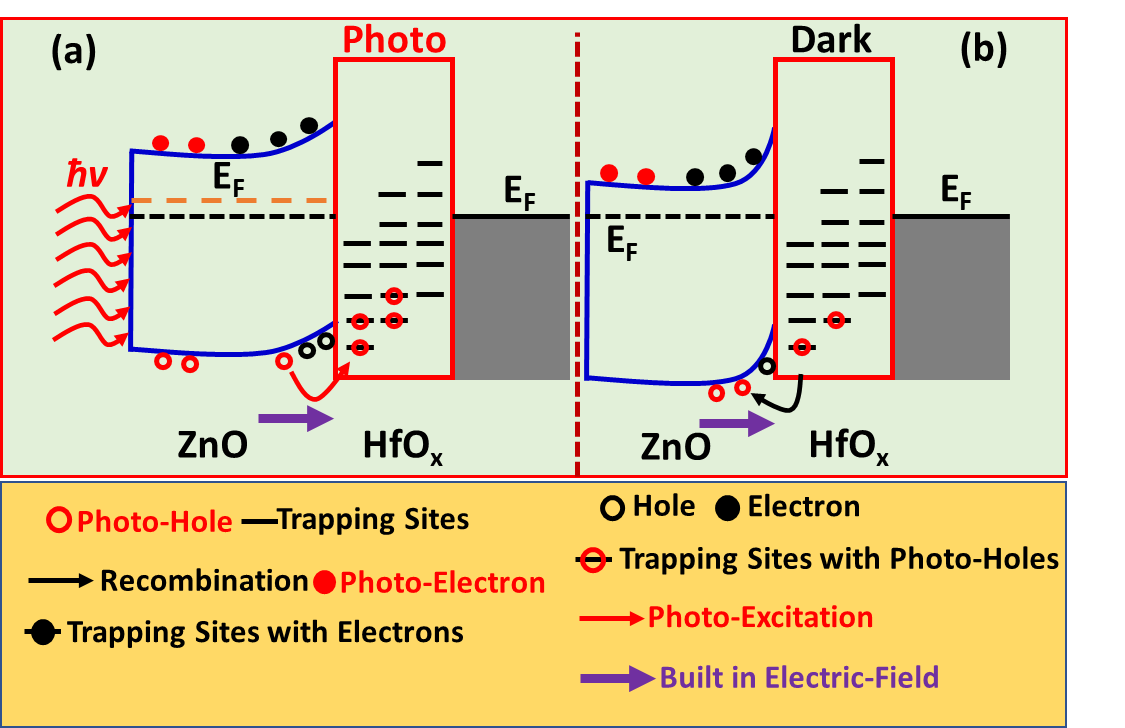


**Fig. S14** Schematic structure of the band diagram of the ZnO/HfO_x_ junction. **a** under light illumination and **b** in dark condition

Based on the bandgap of ZnO and HfO_x_, the valence band (VB) offset between ZnO/HfO_x_ is less than the conduction band (CB) offset [S36, S37]. The mechanism of our optical synapse is described in **Fig. S14**. In the equilibrium condition, a built-in electric field generates at ZnO/HfO_x_ interface due to the different work function of ZnO and HfO_x_ [S31] When optical light is illuminated, photons excite electrons to the conduction band (CB) to contribute to conduction, leaving holes in the valency band (VB). Therefore, the photocurrent of the device is increased (Photonic potentiation). Photogenerated holes are accumulated at the interface and trapped in the HfO_x_ layer under the built-in electric field, which is shown in Figure **S14a**. When the light is turned off, photogenerated holes are returned to the interfacial barrier under thermal excitation (darkness relaxation), as shown in Figure **S14b**. This phenomenon is called persistent photoconductivity.

The energy levels of zinc oxide (ZnO) and hafnium oxide (HfOx) reveal that the valence band offset in the ZnO/HfO_x_ heterojunction is smaller than the conduction band offset. This band alignment plays a crucial role in the photo synaptic effect observed in the current device, as illustrated in **Fig. S14** [S31, S38, S39]. Initially, a built-in electric field is established at the ZnO/HfO_x_ interface due to the difference in work functions between the two materials, indicated by a purple arrow in **Fig. S14a**. Upon exposure to light, photons excite electrons from the valence band to the conduction band of ZnO, increasing the conductivity. Simultaneously, photogenerated holes are created in the valence band and accumulate at the ZnO/HfO_x_ interface. These holes become trapped in the HfO_x_ layer due to the influence of the built-in electric field, as depicted on the left side of **Fig. S14a**. When the light source is removed, the trapped holes can cross the interfacial barrier back into the ZnO layer through thermal excitation. This process is represented schematically in **Fig. S14b**, which illustrates the behavior of the device in the dark. A significant negative voltage applied to the device, as indicated by the blue arrow, results in electrons from the ZnO layer being trapped in deep sites within the HfO_x_ layer. Concurrently, electrons from shallow traps near the electrode migrate into the ITO electrode, contributing to the overall conduction. This detailed mechanism highlights the complex interplay between photogenerated carriers and the built-in electric field at the ZnO/HfO_x_ interface, which is critical for understanding the photo synaptic behavior of the device. The trapping and de-trapping of carriers at the interface and within the HfO_x_ layer are key processes that govern the synaptic-like response, emphasizing the importance of precise control over material properties and interface engineering for optimizing device performance.

**Note-S8 Human Visual Perception System**


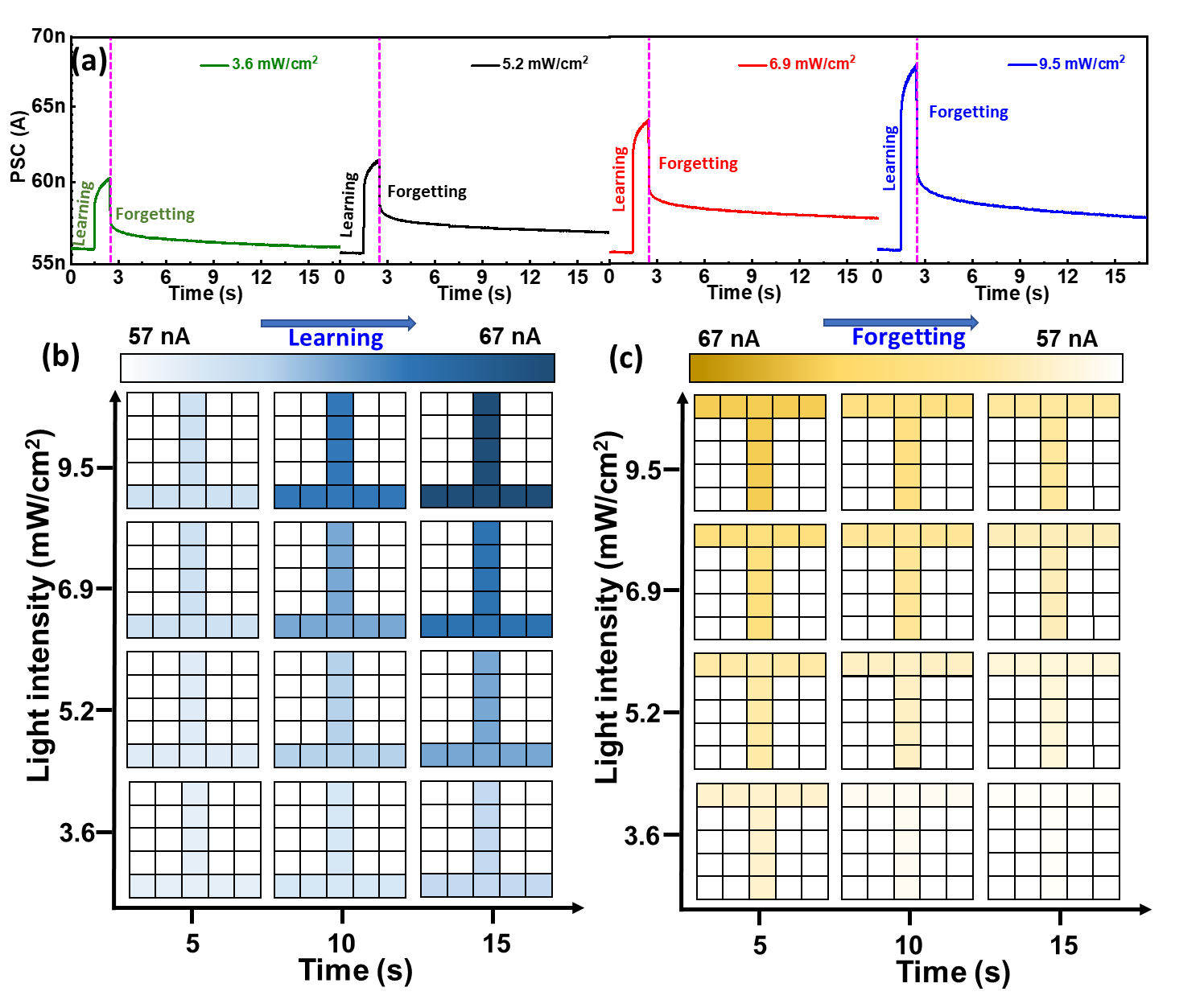
 **Fig. S15** **a** PSC of the device using different light intensities and fixed light exposure time 1 s. **b** Image mapping of the 5×5 array with different light intensities. **c** The conductance mapping to the 5x5 array with various decay times

**Figure S15a** displays the device's temporal response using various light intensities ranging from 3.6 to 9.5 mW/cm^2^. As anticipated, the PSC of the device increases with the intensity of the light, signifying input processing. The PSC gradually declines after turning off the light illumination, depending on the intensity. At a low-intensity level (3.6 mW/cm^2^), the PSC returns to its initial state within 16 seconds. However, for higher intensities, the PSC is sustained at different levels, reflecting distinct memory effects. In fact, enough electron-hole pairs are generated upon light illumination, filling trap states and altering the photo response. Therefore, at higher intensities, more electron-hole pairs are generated, resulting in increased PSC in the device. These findings verify that the device transitions from short-term potentiation (STP) to long-term potentiation (LTP) as light intensity increases, which is analogous to rehearsal in human life. Consequently, the device remembers its programmed state and could be a promising approach for mimicking biological visual perception [S40, S41]. Human visual memory is affected by various factors, such as personal experiences, emotions, and attention, which can influence how information is stored, retrieved, and recalled. Our device exhibits similar optical sensing properties to modulate the light responsivity of visual signals through different light intensities with PSC changes. For the experimental demonstration of our device, 25 devices were randomly selected and used to design a 5x5-pixel array. **Fig. S15b** displays the device's PSC with different light stimuli and times. To achieve information storage and forgetting, we introduced three distinct light intensities (3.6, 5.2, 6.9, and 9.5 mW/cm^2^ through a ⊥-shaped pattern) with varying time durations. A learning process is evident, where the pattern becomes clearer initially as the light intensity increases and pulse duration lengthens. These results suggest that our artificial device perception can reliably memorize image information with stimuli, demonstrating its potential application in perceiving and emulating the vision effect, like human visual perception. Our vision memory also observes a forgetting process with varying decreasing rates (through a T-shaped pattern) but retains some basic information for the long term, as shown in **Fig. S15c**. The device's inherent light sensitivity is suitable for operating and sensing different light intensities and duration times, making it an appropriate candidate for human visual perception emulation.

**Note-S9 Dataset Description**

ECG based arrhythmia detection: We evaluate on the performance of ECG-based arrhythmia detection tasks using MIT-BIH dataset [S42], which comprises recording from a total 47 subjects. It contains forty-eight 30-min excerpts of two-channel ambulatory ECG recordings. These samples were recorded from a mixed collection of inpatients and outpatients with the ration of about 3:2 at Boston’s Beth Israel Hospital. It is trained to classify three abnormal events and one normal event. EMG based hand gesture recognition: To evaluate the performance on EMG based hand gesture recognition task, Nina Pro DB1 benchmark is used in this experiment. Nina Pro DB1 contains surface EMG (sEMG) data collection of 10 electrodes of measurements from 27 subjects [S43]. When collecting data, each type of hand gesture is repeated 10 times in a row with sampling rate of 100 HZ. This experiment's evaluation focused on five commonly used hand gestures: the resting position, the thumb-up position, the middle flexion, the ring flexion, and the index flexion position. The recorded samples were randomly divided into training and testing datasets in a 4:1 ratio, respectively.

EEG based epileptic seizure prediction: To evaluate the performance on EEG based epileptic seizure prediction task, we utilized the CHB-MIT EEG dataset [S44], which comprises 23 scalp EEG recordings from 22 patients at a 256 Hz sampling rate. Data from 15 subjects are recorded based on the fixed 23-electrode configuration, while one or several changes of electrode setting are implemented for the remaining subjects.

**Network Details for Healthcare Applications**

**Table S2** Comparison table of networks for three biosignals


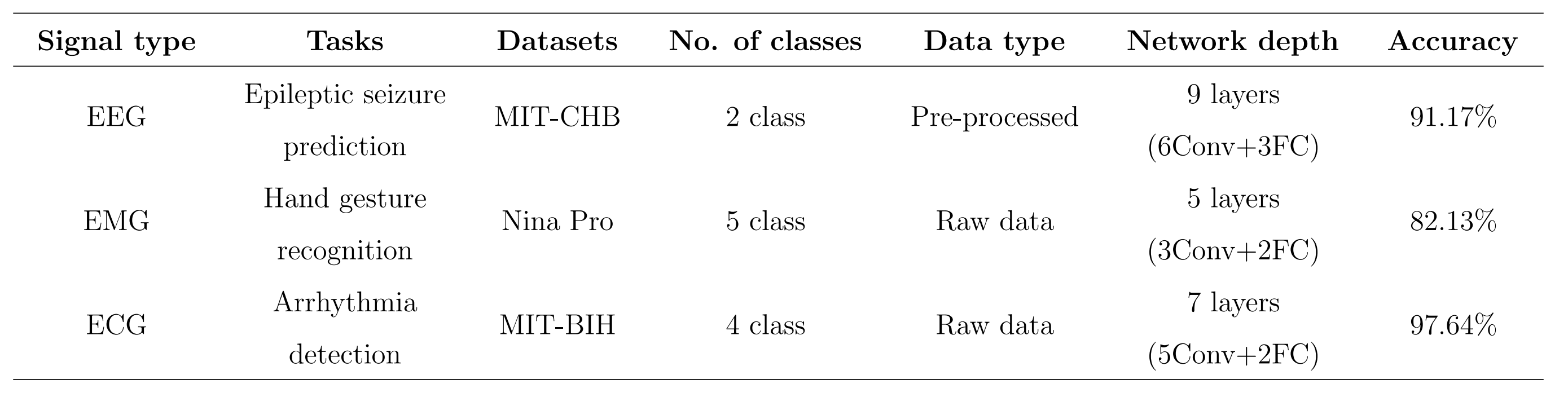


**Note-S10 Process for Fine-tune Process**

**
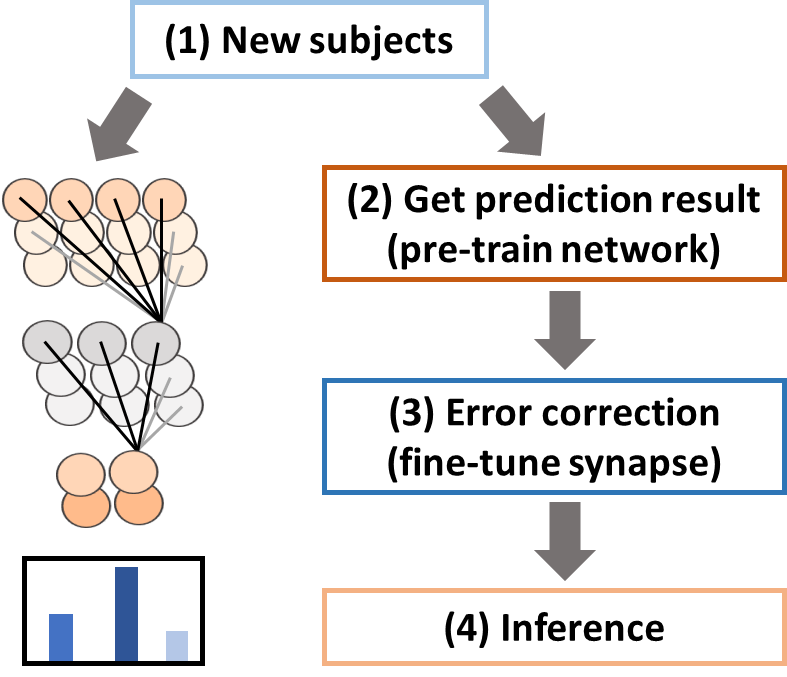
**

**Fig. S16** Process flow for our entire system

**Figure S16** shows the process flow of the proposed method. In the first stage, we pre-train a model based on small samples. When meeting with new subjects or new patients, we fix the weight of convolutional layer but dynamically adjust the weight of fully connected layer through the memristor’s programmability. Finally, the fine-tuned model has a better performance than the original one. In the traditional hardware method, the network weight is permanently fixed after the training process. However, based on the prior knowledge that biomedical features of signals are highly heterogeneous across patients [S45]. Our method focuses on patient-specific manners where one classifier is fine-tuned per subject instead of a universal model for all patients. It requires the hardware fine-tuning process of memristor with the benefits of reconfiguration and programmability. Here is the pseudocode for memristor-based fine-tune process.

Here is the pseudocode for the memristor-based fine-tune process.

**
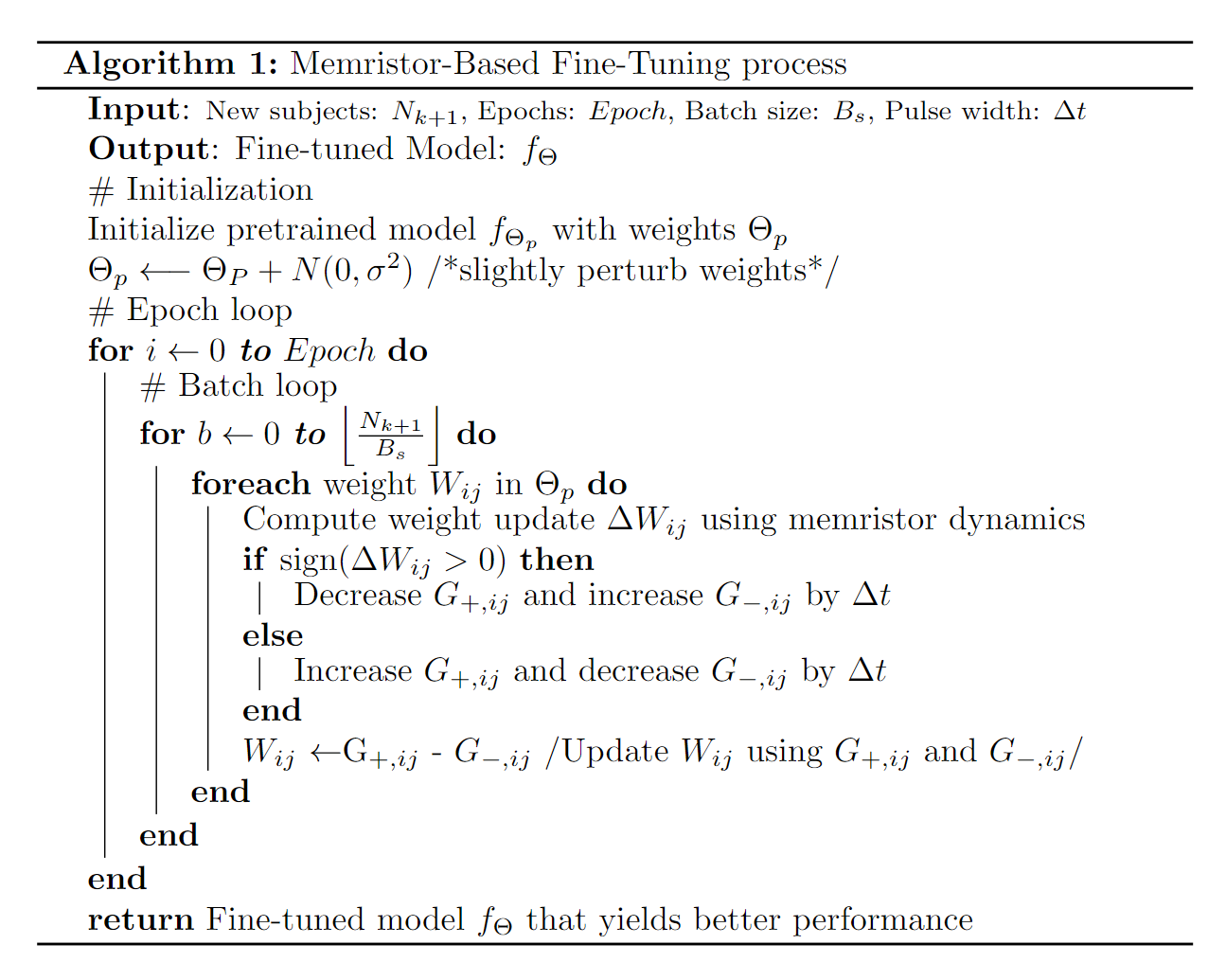
**

**The effect of Device Conductance Variation**

We explore the device conductance variation effect for three biomedical applications. Network accuracy suffers a drop with the increasing of device variation. **Figure S17** shows the classification accuracy across various device conductance variations. We record the minimum and maximum accuracy during 10 training iterations. With higher device conductance variation, the network accuracy shows a wider range of accuracy with decrease of training accuracy.


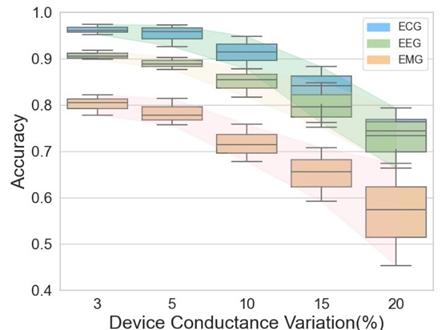


**Fig. S17** The effects of device conductance variation on the classification accuracy. The device variation varies from 3% to 20%

**Supplementary References**

1. A. Liang, J. Zhang, F. Wang, Y. Jiang, K. Hu et al., Transparent HfOx-based memristor with robust flexibility and synapse characteristics by interfacial control of oxygen vacancies movement. Nanotechnology **32**, 145202 (2021). <https://doi.org/10.1088/1361-6528/abd3c7>
2. S. Kim, Y. Abbas, Y. R. Jeon, A. S. Sokolov, B. Ku et al., Engineering synaptic characteristics of TaOx/HfO2 bi-layered resistive switching device. Nanotechnology **29**, 415204 (2018). <https://doi.org/10.1088/1361-6528/aad64c>
3. Y. Sun, J. Wang, D. He, M. Yang, C. Jiang et al., Enhanced resistive switching uniformity in HfO_2_/TiO_2_ NWA memristor for synaptic simulation. Appl. Phys. Lett. **122**, 133501 (2023). <https://doi.org/10.1063/5.0140746>
4. S. Biswas, A. D. Paul, P. Das, P. Tiwary, H. J. Edwards et al., Impact of AlOy interfacial layer on resistive switching performance of flexible HfOₓ/AlOy ReRAMs. IEEE Trans. Electron Devices **68**, 3787-3793 (2021). <https://doi.org/10.1109/TED.2021.3084554>
5. W. Zhang, Z. Guo, Y. Dai, J. Lei, J. Wang, Effects of stacking sequence and top electrode configuration on switching behaviors in ZnO-HfO2 hybrid resistive memories. Ceram. Int. **49**, 35973-35981 (2023). <https://doi.org/10.1016/j.ceramint.2023.08.277>
6. A. Saleem, D. Kumar, A. Singh, S. Rajasekaran, T.Y. Tseng, Oxygen vacancy transition in HfOx-based flexible, robust, and synaptic Bi-layer memristor for neuromorphic and wearable applications. Adv. Mater. Technol. **7**, 2101208 (2022). <https://doi.org/10.1002/admt.202101208>
7. Y. Yang, X. Zhu, Z. Ma, H. Hu, T. Chen, Artificial HfO_2_/TiO_x_ synapses with controllable memory window and high uniformity for brain-inspired computing. Nanomaterials **13**, 605 (2023). <https://doi.org/10.3390/nano13030605>
8. S. Chandrasekaran, F. M. Simanjuntak, D. Panda, T.Y. Tseng, Enhanced synaptic linearity in ZnO-based invisible memristive synapse by introducing double pulsing scheme. IEEE Trans. Electron Devices **66**, 4722-4726 (2019). <https://doi.org/10.1109/TED.2019.2941764>
9. Y. Wang, G. Zhou, B. Sun, W. Wang, J. Li et al., Ag/HfOx/Pt unipolar memristor for high-efficiency logic operation. J. Phys. Chem. Lett. **13**, 8019-8025 (2022). <https://doi.org/10.1021/acs.jpclett.2c01906>
10. M. Kumar, S. Abbas, J. Kim, All-oxide-based highly transparent photonic synapse for neuromorphic computing. ACS Appl. Mater. Interfaces **10**, 34370-34376 (2018). <https://doi.org/10.1021/acsami.8b10870>
11. P. X. Chen, D. Panda, T. Y. Tseng, All oxide based flexible multi-folded invisible synapse as vision photo-receptor. Sci. Rep. **13**, 1454 (2023). | <https://doi.org/10.1038/s41598-023-28505-3>
12. D.C. Hu, R. Yang, L. Jiang, X. Guo, Memristive synapses with photoelectric plasticity realized in ZnO_1–x_/AlO_y_ heterojunction. ACS Appl. Mater. Interfaces **10**, 6463-6470 (2018). <https://doi.org/10.1021/acsami.8b01036>
13. Z. Zhou, Y. Pei, J. Zhao, G. Fu, X. Yan, Visible light responsive optoelectronic memristor device based on CeOx/ZnO structure for artificial vision system. Appl. Phys. Lett. **118**, 191103 (2021). <https://doi.org/10.1063/5.0047403>
14. M. Noh, D. Ju, S. Cho, S. Kim, The enhanced performance of neuromorphic computing hardware in an ITO/ZnO/HfOx/W bilayer-structured memory device. Nanomaterials **13**, 2856 (2023). <https://doi.org/10.3390/nano13212856>
15. S. Zhu, B. Sun, G. Zhou, T. Guo, C. Ke et al., In-depth physical mechanism analysis and wearable applications of HfOx-based flexible memristors. ACS Appl. Mater. Interfaces **15**, 5420-5431 (2015). <https://doi.org/10.1021/acsami.2c16569>
16. P. Pal, K. J. Lee, S. Thunder, S. De, P. T. Huang et al., Bending resistant multibit memristor for flexible precision inference engine application. IEEE Trans. Electron Devices **69**, 4737-4743 (2022). <https://doi.org/10.1109/TED.2022.3186965>
17. D. Kumar, L.B. Keong, N. E. Atab, T.Y. Tseng, Enhanced synaptic features of ZnO/TaOx bilayer invisible memristor for brain-inspired computing. IEEE Electron Device Lett. **43**, 2093-2096 (2022). <https://doi.org/10.1109/LED.2022.3217983>.
18. C. L. Hsu, A. Saleem, A. Singh, D. Kumar, T.Y. Tseng, Enhanced linearity in CBRAM synapse by post oxide deposition annealing for neuromorphic computing applications. IEEE Trans. Electron Devices **68**, 5578-5584 (2021). <https://doi.org/10.1109/TED.2021.3112109>
19. H. Jiang, L. Han, P. Lin, Z. Wang, M. H. Jang et al., Sub-10 nm Ta channel responsible for superior performance of a HfO_2_ memristor. Sci. Rep. **6**, 28525 (2016). <https://doi.org/10.1038/srep28525>
20. L. Liu, W. Xiong, Y. Liu, K. Chen, Z. Xu, Designing high-performance storage in HfO_2_/BiFeO_3_ memristor for artificial synapse applications. Adv. Electron. Mater. **6**, 1901012 (2020). <https://doi.org/10.1002/aelm.201901012>
21. P. Subin, P. Midhun, A. Antony, K. Saji, M. Jayaraj, Optoelectronic synaptic plasticity mimicked in ZnO-based artificial synapse for neuromorphic image sensing application. Mater. Today Commun. **33**, 104232 (2022). <https://doi.org/10.1016/j.mtcomm.2022.104232>
22. R. Zhang, H. Huang, Q. Xia, C. Ye, X. Wei et al., Role of oxygen vacancies at the TiO_2_/HfO_2_ interface in flexible oxide-based resistive switching memory. Adv. Electron. Mater. **5**, 1800833 (2019). <https://doi.org/10.1002/aelm.201800833>
23. N. Jain, S. K. Sharma, R. Kumawat, P. K. Jain, D. Kumar et al., Resistive switching, endurance and retention properties of ZnO/HfO_2_ bilayer heterostructure memory device. Micro Nanostructures **169**, 207366 (2022). <https://doi.org/10.1016/j.micrna.2022.207366>
24. X. Shan, C. Zhao, Y. Lin, J. Liu, X. Zhang et al., Optoelectronic synaptic device based on ZnO/HfOx heterojunction for high-performance neuromorphic vision system. Appl. Phys. Lett. **121**, 263501 (2022). <https://doi.org/10.1063/5.0129642>
25. L. Ma, G. Wang, S. Wang, D. Chen, Simulation of in-situ training in spike neural network based on non-ideal memristors. IEEE J. Electron Devices Soc. **11**, 497-502 (2023). <https://doi.org/10.1109/JEDS.2023.3311763>
26. S. Shrivastava, L.B. Keong, S. Pratik, A.S. Lin, T.Y. Tseng, Fully photon controlled synaptic memristor for neuro-inspired computing. Adv. Electron. Mater. **9**, 2201093 (2023). <https://doi.org/10.1002/aelm.202201093>
27. C. L. Hsu, A. Saleem, A. Singh, D. Kumar, T.Y. Tseng, Enhanced linearity in CBRAM synapse by post oxide deposition annealing for neuromorphic computing applications. IEEE Trans. Electron Devices **68**, 5578-5584 (2021). <https://doi.org/10.1109/TED.2021.3112109>
28. T.-L. Tsai, H.-Y. Chang, F.-S. Jiang, and T.-Y. Tseng, Impact of post-oxide deposition annealing on resistive switching in HfO_2_-based oxide rram and conductive-bridge RAM devices. IEEE Electron Device Lett*.* **36**, 1146 (2015). <https://doi.org/10.1109/LED.2015.2477491>
29. R. Zhang, H. Huang, Q. Xia, C. Ye, X. Wei et al., Role of Oxygen vacancies at the TiO_2_/HfO_2_ interface in flexible oxide-based resistive switching memory. Adv. Electron. Mater. **5**, 1800833 (2019). <https://doi.org/10.1002/aelm.201800833>
30. K. M. Kim, S. J. Song, G. H. Kim, J. Y. Seok, M. H. Lee et al., Collective motion of conducting filaments in Pt/n-Type TiO_2_/p-Type NiO/Pt stacked resistance switching memory. Adv. Funct. Mater. **21**, 1987 (2011). <https://doi.org/10.1002/adfm.201002282>
31. [J. B. Roldán](https://onlinelibrary.wiley.com/authored-by/Rold%C3%A1n/Juan+B.), [E. Miranda](https://onlinelibrary.wiley.com/authored-by/Miranda/Enrique), [D. Maldonado](https://onlinelibrary.wiley.com/authored-by/Maldonado/David), [A. N. Mikhaylov](https://onlinelibrary.wiley.com/authored-by/Mikhaylov/Alexey+N.), [N. V. Agudov](https://onlinelibrary.wiley.com/authored-by/Agudov/Nikolay+V.) et al., Variability in resistive memories. Adv. Intell. Syst. **5**, 2200338 (2023). <https://doi.org/10.1002/aisy.202200338>
32. J. Yang, A. Yoon, D. Lee, S. Song, I. L. J. Jung et al., Wafer-scale memristor array based on aligned grain boundaries of 2D molybdenum ditelluride for application to artificial synapses. Adv. Funct. Mater. **34**, 2309455 (2024). <https://doi.org/10.1002/adfm.202309455>
33. M. Lanza, F. Hui, C. Wen, A. C. Ferrar, Resistive switching crossbar arrays based on layered materials. Adv. Mater. **35**, 2205402 (2023). <https://doi.org/10.1002/adma.202205402>
34. M. Lanza, R.Waser, D. Ielmini, J. J.Yang, L.Goux et al., Standards for the characterization of endurance in resistive switching devices. ACS Nano **15**, 17214–17231 (2021). <https://doi.org/10.1021/acsnano.1c06980>
35. Z. Ma, J. Ge, W. Chen, X. Cao, S. Diao et al., Reliable memristor based on ultrathin native silicon oxide. ACS Appl. Mater. Interfaces **14**, 21207–21216 (2022). <https://doi.org/10.1021/acsami.2c03266>
36. D. C. Hu, R. Yang, L. Jiang, and X. Guo, Memristive synapses with photoelectric plasticity realized in ZnO_1–x_/AlO_y_ heterojunction. ACS Appl. Mater. Interfaces **10**, 6463-6470 (2018). <https://doi.org/10.1021/acsami.8b01036>
37. U. Chand, K. C. Huang, C.Y. Huang, and T. Y. Tseng, Mechanism of nonlinear switching in HfO_2_-based crossbar RRAM with inserting large bandgap tunneling barrier layer. IEEE Trans. Electron Devices **62**, 3665 (2015). <https://doi.org/10.1109/TED.2015.2471835>
38. H.Tan, G. Liu, X. Zhu, H.Yang, B. Chen, X. Chen et al., An optoelectronic resistive switching memory with integrated demodulating and arithmetic functions. Adv. Mater. **27**, 2797–2803 (2015). <https://doi.org/10.1002/adma.201500039>
39. H. Tan, G. Liu, H. Yang, X. Yi, L. Pan et al., Light-gated memristor with integrated logic and memory functions. ACS Nano **11**, 11298-11305 (2017). <https://doi.org/10.1021/acsnano.7b05762>
40. F. Zhou, Z. Zhou, J. Chen, T. H. Choy, J. Wang et al., Optoelectronic resistive random access memory for neuromorphic vision sensors. Nat. Nanotechnol*.* **14**, 776-782 (2019). <https://doi.org/10.1038/s41565-019-0501-3>
41. J. Chen, Z. Zhou, B. J. Kim, Y. Zhou, Z. Wang et al., Optoelectronic graded neurons for bioinspired in-sensor motion perception. Nat. Nanotechnol*.* **18**, 882 (2023). <https://doi.org/10.1038/s41565-023-01379-2>
42. A.L. Goldberger, L.A. Amaral, L. Glass, J.M. Hausdorff, P.C. Ivanov et al., Components of a new research resource for complex physiologic signals. Circulation **101**, e215-e220 (2000). <https://doi.org/10.1161/01.CIR.101.23.e215>
43. M. Atzori, A. Gijsberts, C. Castellini, B. Caputo, A. G. M. Hager et al., Electromyography data for non-invasive naturally-controlled robotic hand prostheses. Scientific data **1**, 140053 (2014). <https://doi.org/10.1038/sdata.2014.53>
44. S.A. Hossam, Diss. *Massachusetts Institute of Technology*, **2009**.
45. The causes of epilepsy: common and uncommon causes in adults and children [M]. *Cambridge University Press*, **2011**.
